# Benzylisoquinoline Alkaloid Production in Yeast via Norlaudanosoline Improves Titer, Selectivity, and Yield

**DOI:** 10.1021/acssynbio.5c00897

**Published:** 2026-03-04

**Authors:** Lauren Narcross, Michael E. Pyne, Kaspar Kevvai, Ka-Hei Siu, John E. Dueber, Vincent J. J. Martin

**Affiliations:** † Department of Biology, 5618 Concordia University, Montréal, Québec H4B 1R6, Canada; ‡ Centre for Applied Synthetic Biology, Concordia University, Montréal, Québec H4B 1R6, Canada; § Department of Bioengineering, 1438 University of California, Berkeley, Berkeley, California 94720, United States; ∥ Biological Systems & Engineering Division, Lawrence Berkeley National Laboratory, Berkeley, California 94720, United States

**Keywords:** metabolic engineering, synthetic biology, benzylisoquinoline alkaloids, reticuline, *Saccharomyces cerevisiae*, yeast

## Abstract

The benzylisoquinoline alkaloid (BIA) family of tetrahydroisoquinolines (THIQs) comprises >2,500 members, including the pharmaceuticals morphine, codeine, and papaverine, as well as the antibiotics sanguinarine and chelerythrine. Agricultural cultivation can supply the demand for the BIAs that accumulate in plants, but broader access to the BIA family would facilitate additional research and commercialization. Microbial synthesis presents an attractive option due to cheap feedstock, genetic tractability, and ease of scale-up. Previously, we reported titers of the branch-point BIA (*S*)-reticuline of 4.6 g/L in yeast, which was achieved through leveraging the Ehrlich pathway 2-oxoacid decarboxylase Aro10 to generate the intermediate 4-hydroxyphenylacetaldehyde (4-HPAA). Here, we establish a superior route to (*S*)-reticuline by switching the pathway intermediate from 4-HPAA to 3,4-dihydroxyphenylacetaldehyde (3,4-dHPAA) using monoamine oxidase A (MAO). The resulting (*S*)-norlaudanosoline route to (*S*)-reticuline synthesis is more selective, resolving prior issues with off-pathway THIQs synthesized due to cascading enzyme promiscuity, and more efficient, enabling titers of 4.8 g/L (*S*)-reticuline while improving yields by over 40%, from 17 to 24 mg/g sucrose in fed-batch fermentations. Finally, we extend *de novo* (*S*)-reticuline synthesis to dihydrosanguinarine, achieving 635 mg/L dihydrosanguinarine and sanguinarine in fed-batch fermentation, the highest reported titer of these BIAs by a factor of 40.

## Graphical abstract

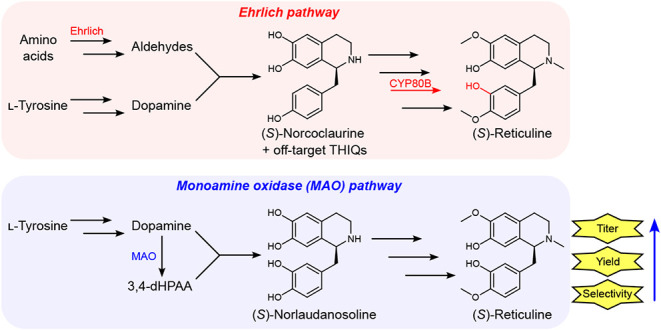

## Introduction

Benzylisoquinoline alkaloids (BIAs) are a large class of plant secondary metabolites in the tetrahydroisoquinoline (THIQ) family with broad applications in human health and agriculture. While some BIAs accumulate to a sufficient degree in plants to allow for commercial-scale production, most do not. A sustainable, scalable source of BIAs would expand access to this valuable class of natural products. One promising option is the introduction of the BIA synthesis to a microbial host.

BIA synthesis from simple carbon sources has been established in *Escherichia coli*,^1^ *Saccharomyces cerevisiae*,^2,3^ and *Pichia pastoris*.^4^ (*S*)-Reticuline is a common target for *de novo* BIA synthesis, as it is the last shared pathway intermediate in the morphine, sanguinarine, and noscapine pathways.^5^ To date, the highest reported (*S*)-reticuline titers are 8 mg/L in *P. pastoris*,^4^ 0.16 g/L in *E. coli*,^6^ and 4.6 g/L in *S. cerevisiae*.^7^ The latter titer is noteworthy, because it approaches a target set for the commercial production of opioids in microbes: 5 g/L.^8^ Compared to bacterial and alternative yeast hosts (e.g., *P. pastoris*), *S. cerevisiae* has enabled substantially higher titers and scalability, in part due to its compatibility with plant P450 enzymes, its rapid and sophisticated strain engineering toolkit, and the ability to deregulate (e.g., Aro3^K222L^, Aro4^K229L^, and Aro7^G141S^) and overexpress the shikimate and l-tyrosine precursor pathways. Beginning with the pioneering demonstration of *de novo* opioid biosynthesis in yeast,^8^ *S. cerevisiae* has been engineered to produce an array of plant BIAs from sugar, including noscapine, berberine, sanguinarine, pronuciferine, tetrahydropapaverine, and several high-molecular weight bis-BIAs.^9–13^ BIA biosynthesis in native plants and engineered microorganisms has been reviewed recently.^14–17^

The committed step of BIA synthesis in plants is the formation of (*S*)-norcoclaurine by norcoclaurine synthase (NCS) through the condensation of dopamine and 4-hydroxyphenylacetaldehyde (4-HPAA), both derivatives of the aromatic amino acid pathway (Figure 1A).^5^ In yeast, dopamine is a non-native metabolite, requiring the expression of heterologous enzymes for the hydroxylation and decarboxylation of l-tyrosine. 4-HPAA is a native yeast metabolite, derived from the tyrosine precursor 4-hydroxyphenylpyruvate (4-HPP) by the 2-oxoacid decarboxylase Aro10 as part of the Ehrlich pathway of amino acid catabolism.^18,19^ While overexpression of *ARO10* is an effective choice for 4-HPAA overproduction in yeast (Figure 1A),^7,9^ the enzyme is also capable of synthesizing analogous aldehydes from the 2-oxoacid precursors of the l-amino acids phenylalanine, tryptophan, methionine, leucine, isoleucine, and valine.^20,21^ Compounding the effects of this promiscuity, NCS can condense dopamine with a variety of aldehydes in addition to 4-HPAA, leading to the synthesis of additional THIQs, which are in turn substrates for BIA *O*- and *N*-methyltransferases.^22,23^ Although this web of overlapping promiscuities can enable the elucidation and establishment of synthetic routes to new compounds,^7^ a more selective mechanism for aldehyde synthesis would further improve targeted BIA production in yeast.

**Figure 1:**
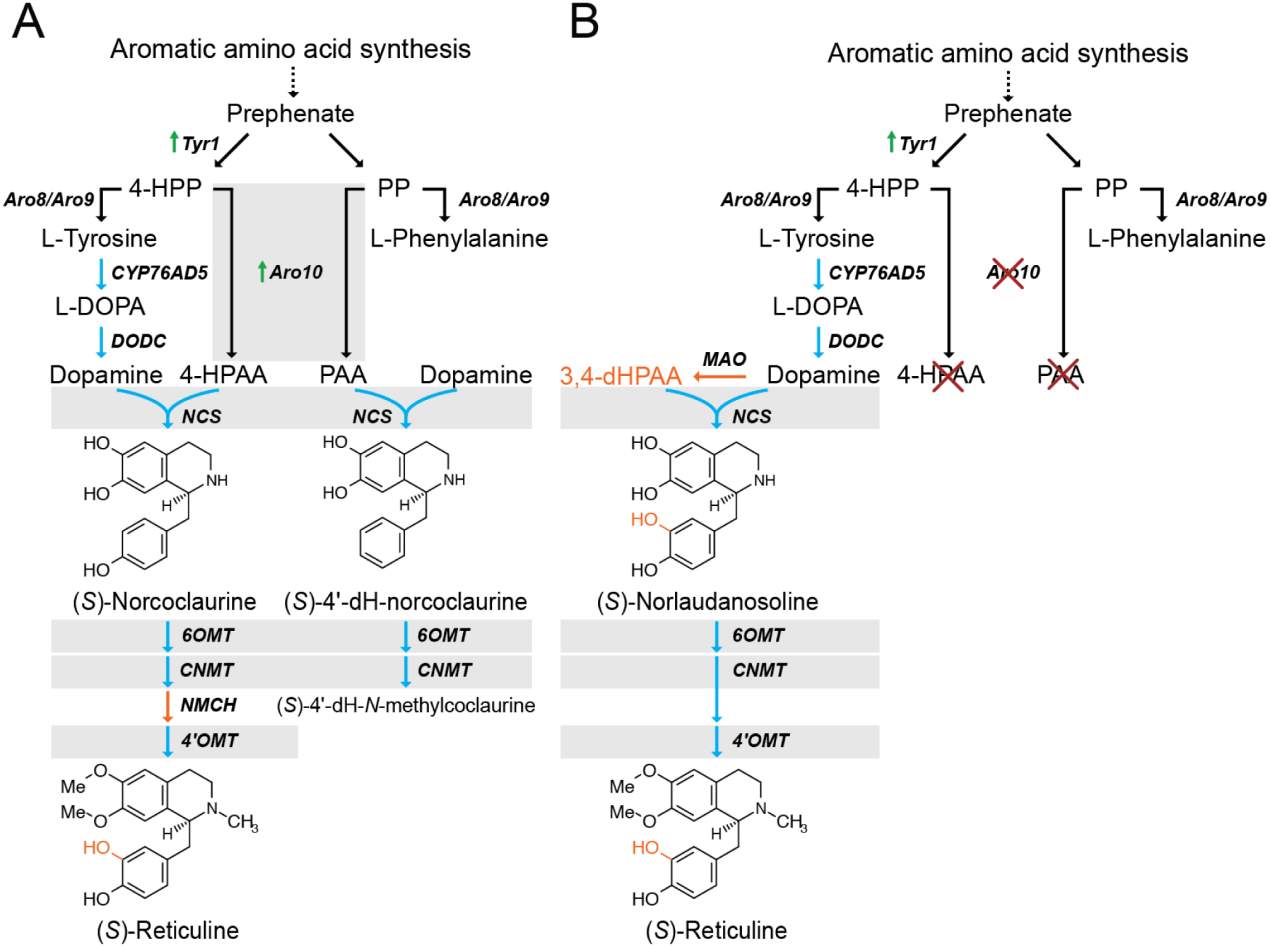
Two pathways for (*S*)-reticuline synthesis in yeast. (A) (*S*)-Reticuline synthesis in yeast proceeding through (*S*)-norcoclaurine. NCS catalyzes the condensation of dopamine and 4-HPAA to generate (*S*)-norcoclaurine, which is methylated and hydroxylated to form (*S*)-reticuline. Due to the promiscuity of multiple pathway enzymes (gray boxes), (*S*)-4́-dehydronorcoclaurine and methylated derivatives are also produced when Aro10 is used to synthesize 4-HPAA. (B) (*S*)-Reticuline synthesis in yeast proceeding through (*S*)-norlaudanosoline, using largely the same enzymes as the (*S*)-norcoclaurine route. The key difference is the synthesis of the aldehyde 3,4-dHPAA from dopamine by MAO. The additional hydroxyl group on 3,4-dHPAA bypasses the requirement for NMCH-catalyzed hydroxylation. The two routes to (*S*)-reticuline’s 3́ hydroxyl group are highlighted in orange. Yeast native enzymes are indicated with black arrows; heterologous enzymes are indicated with blue arrows. Compound abbreviations: PEP, phosphoenolpyruvate; E4P, erythrose 4-phosphate; DHAP, 2-dehydro-3-deoxy-D-arabino-heptonoate 7-phosphate; 4-HPP, 4-hydroxyphenylpyruvate; 4-HPAA, 4-hydroxyphenylacetaldehyde; PP, phenylpyruvate; PAA, phenylacetaldehyde; 3,4-dHPAA, 3,4-dihydroxyphenylacetaldehyde. Enzyme abbreviations: DODC, l-DOPA decarboxylase; NCS, norcoclaurine synthase; MAO, monoamine oxidase; NMCH, *N*-methylcoclaurine hydroxylase; 6OMT, norcoclaurine 6-*O*-methyltransferase; CNMT, coclaurine *N*-methyltransferase; 4́OMT, 3́-hydroxyl-*N*-methylcoclaurine 4́ O-methyltransferase.

Synthesis of 4-HPAA from l-tyrosine through the intermediate tyramine circumvents the need for *ARO10* overexpression. Corresponding enzymes have been identified^24^ and expressed in yeast,^25^ resulting in 0.08 g/L BIA synthesis in shake-flasks. An alternative 4-HPAA-independent route to (*S*)-reticuline leverages 3,4-dihydroxyphenylacetaldehyde (3,4-dHPAA) to form (*S*)-norlaudanosoline. This pathway is largely similar to that of the (*S*)-norcoclaurine route; however, (*S*)-norlaudanosoline synthesis bypasses the requirement for the downstream cytochrome P450-catalyzed hydroxylation of *N*-methylcoclaurine due to the presence of a second hydroxyl group on 3,4-dHPAA (Figure 1B, orange highlights). Importantly, production of norlaudanosoline via 3,4-dHPAA does not involve the yeast Ehrlich pathway, which could be inactivated to prevent the synthesis of off-target THIQs,^7^ thus improving BIA titer and selectivity. This has made norlaudanosoline the preferred route for BIA production in *E. coli*, in which several enzymes synthesizing 3,4-dHPAA from either l-DOPA^26^ or dopamine have been used. The highest titers (0.16 g/L reticuline) were achieved through the dopamine route, using monoamine oxidase from *Micrococcus luteus* (*Ml*MAO).^6^ Although *Ml*MAO was identified as a major bottleneck in *de novo* BIA synthesis in *E. coli*,^6^ the success of the norlaudanosoline route for aldehyde synthesis highlights an opportunity to improve specificity and productivity of BIA production in yeast.

In this work, a yeast strain engineered to synthesize (*S*)-reticuline via (*S*)-norcoclaurine was retrofitted to synthesize (*S*)-reticuline via (*S*)-norlaudanosoline. 3,4-dHPAA synthesis from dopamine was achieved using human monoamine oxidase A (*Hs*MAO-A, hereafter MAO). The (*S*)-norlaudanosoline route to BIA synthesis in yeast enabled higher (*S*)-reticuline titers compared to the (*S*)-norcoclaurine route at a higher yield while almost eliminating undesirable condensation products. The heterologous pathway was further extended to the benzophenanthridines dihydrosanguinarine and sanguinarine, resulting in a titer of 653 mg/L in fed-batch fermentation—surpassing previous microbially produced titers 40-fold.^25^

## Results

### Additional Copies of BIA Modifying Genes Reduce Accumulation of Pathway Intermediates

Previously, we reported *de novo* synthesis of benzylisoquinoline alkaloids (BIAs) in yeast at gram-per-liter scale, with the final strain (LP507) reaching 4.6 g/L (*S*)-reticuline in fed-batch fermentations with pulsed sugar feeding.^7^ However, mass spectrometry analyses revealed that in addition to (*S*)-reticuline, strain LP507 also produced considerable quantities of other tetrahydroisoquinolines (THIQs). Based on the total ion count, (*S*)-reticuline accounted for only 42% of all THIQs present in the final fermentation broth (Figure 2A and B). An additional 25% was attributable to other benzyl-THIQ BIA pathway intermediates upstream of (*S*)-reticuline, namely (*S*)-norcoclaurine, (*S*)-coclaurine, (*S*)-*N*-methylcoclaurine, and (*S*)-3**´**-hydroxy-*N*-methylcoclaurine (hereafter referred to as “other benzyl-THIQs”). The final 33% corresponded to THIQs resulting from the condensation of dopamine with phenylacetaldehyde (PAA) derived from the catabolism of phenylalanine (hereafter referred to as “4′-unsubstituted benzyl-THIQs”).

**Figure 2:**
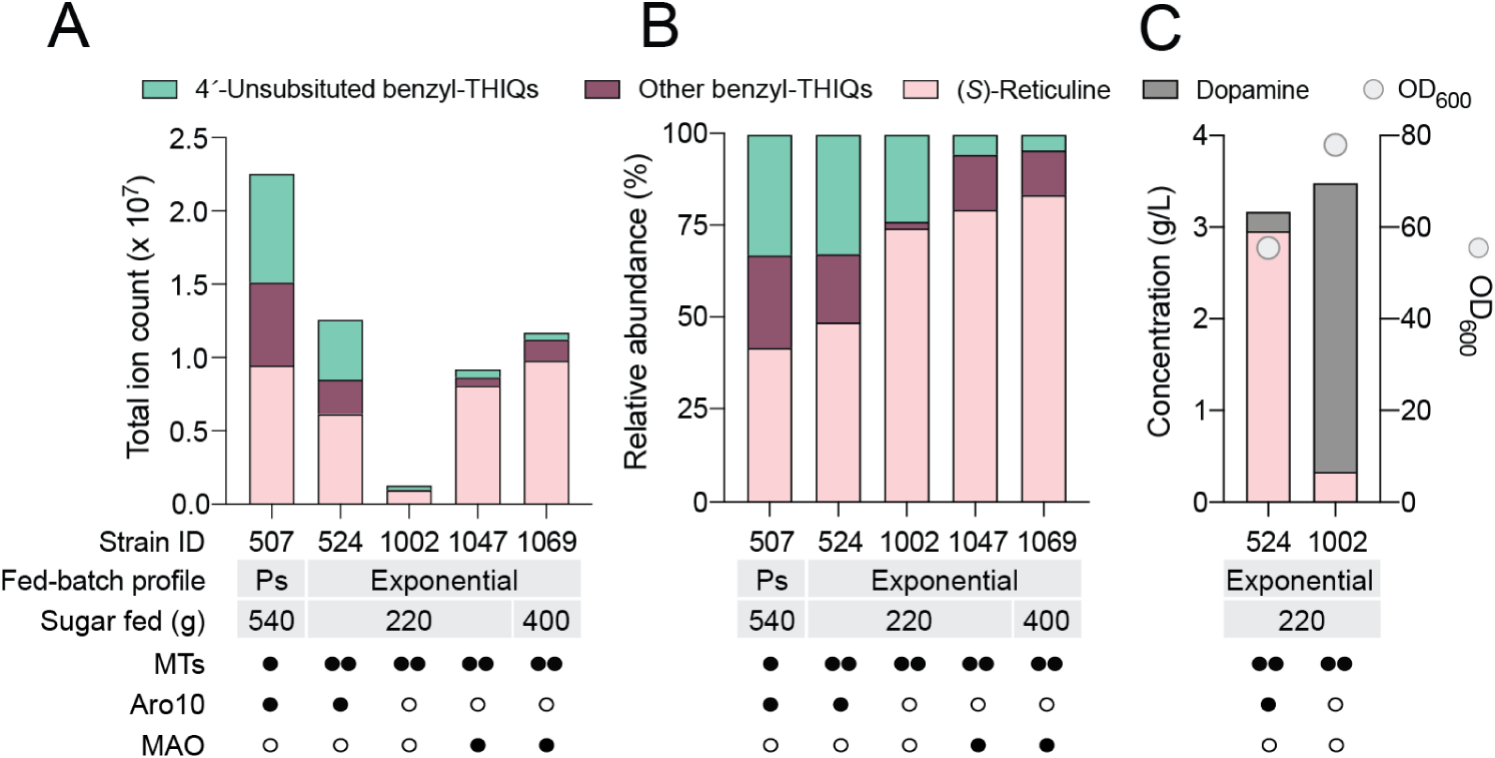
Effects of *ARO10* knockout in yeast synthesizing benzylisoquinoline alkaloids in fed-batch fermentation. (A) Abundance of THIQs in select fed-batch fermentations. Sugar feeding was carried out either as a series of pulses controlled by off-gas analysis (Ps) or exponentially at a preset constant growth rate. End-point samples were analyzed for (*S*)-reticuline, benzyl-THIQ pathway intermediates, and off-pathway 4́-unsubstituted benzyl-THIQs originating from the condensation of dopamine and PAA. (B) Relative abundance of THIQs in the same fed-batch fermentations. (C) Impact of *ARO10* knockout on dopamine, (*S*)-reticuline, and biomass concentrations. The native and heterologous copies of *ARO10* were deleted from strain LP524 (strain LN1002), and both strains were grown in fed-batch fermentation with the same media and feeding profile. End-point samples were analyzed for OD_600_ and metabolite content. Symbols: ○ no enzyme; • one enzyme copy; •• two copies. Abbreviations: MTs, methyltransferases; MAO, monoamine oxidase.

To improve the (*S*)-reticuline synthesis, we first sought to reduce the abundance of other benzyl-THIQs. Additional copies of pathway genes *6OMT*, *CNMT*, and *NMCH* were introduced into LP507, resulting in strain LP524. Analysis of total THIQs from fed-batch fermentation of LP524 with an exponential sugar feeding regimen revealed that the relative abundance of other benzyl-THIQs fell from 25% to 18% (Figure 2B, strain LP524). However, 4′-unsubstituted benzyl-THIQs still comprised 33% of the total peak area, indicating that further efforts should be focused on improving the selectivity of THIQ synthesis.

### *ARO10* Knockout Minimizes *De Novo* 4́-Dehydroxynorcoclaurine Synthesis

The dominant off-pathway side products detected in strain LP524 were 4́-unsubstituted benzyl-THIQs derived from the condensation of dopamine with PAA, specifically 4́-dehydroxynorcoclaurine (4**´**-dHN) and its methylated derivatives 4**´**-dehydroxycoclaurine and 4**´**-dehydroxy-*N*-methylcoclaurine (Figure 1A and Figure 2). NCS has a high degree of promiscuity for aldehydes, and will efficiently condense PAA with dopamine to produce 4**´**-dHN.^22,23^ Therefore, to limit 4́-unsubstituted benzyl-THIQ formation, the source of PAA synthesis in BIA-producing yeast should be identified and eliminated. Several 2-oxoacid decarboxylases have been identified in yeast,^21^ with Aro10 acting as the major decarboxylase contributing to PAA synthesis (Figure 1A).^18,19^ As *ARO10* was overexpressed in LP507 and LP524, we hypothesized that this enzyme was responsible for high levels of PAA production in our strains.

To test this hypothesis, both the native and overexpressed copies of *ARO10* were deleted in strain LP524 to generate strain LN1002. Strains LP524 and LN1002 were both grown in fed-batch fermentations using the same feeding profile and amount of sugar. Under these conditions, LP524 synthesized 3.0 g/L of (*S*)-reticuline and 0.2 g/L dopamine, whereas *aro10*Δ*L*N1002 synthesized 3.1 g/L of dopamine and 0.33 g/L (*S*)-reticuline (Figure 2C). The 90% reduction in (*S*)-reticuline levels demonstrates the dominant contribution of Aro10 to 4-HPAA synthesis in the parent strain. Despite the complete removal of Aro10 from the strain, a small amount of (*S*)-reticuline remains due to the activity of remaining pyruvate decarboxylases, such as Pdc5^18^. Importantly, mass spectrometry revealed almost complete ablation of 4́-unsubstituted benzyl-THIQs (Figure 2A and B), confirming that Aro10 was also largely responsible for PAA synthesis in LP524. Eliminating Aro10 activity also improved biomass accumulation in the bioreactor; strain LN1002 grew to a final OD_600_ of 78 compared to 55 for strain LP524, likely due to reduced accumulation of aldehydes derived from Aro10-mediated catabolism of tyrosine, phenylalanine, tryptophan, and leucine.^7^

### Expression of *MAO* in an *ARO10* Knockout Strain Restores BIA Synthesis

With high dopamine titers and low off-target metabolite accumulation, the *aro10*Δ*L*N1002 strain provided a clean starting point for studying an alternative route to aldehyde generation for BIA synthesis. We opted to explore the production of 3,4-dHPAA, which can be synthesized from and subsequently condensed with dopamine to form the norcoclaurine analog norlaudanosoline (Figure 1B). We chose to examine human MAO (*Hs*MAO), which was previously suggested as a candidate for (*S*)-norlaudanosoline synthesis in yeast.^27^

First, we determined the impact of promoter strength of MAO on (*S*)-reticuline synthesis and strain fitness in the microtiter plate format. *MAO* codon-optimized for expression in yeast was introduced to LN1002 under four well-characterized promoters spanning several orders of magnitude of relative strength.^28^ MAO activity was assessed by growing strains in deep-well plates and measuring their metabolite profile. *MAO* expression reduced dopamine titers in LN1002 in a promoter strength-dependent manner (Figure 3A). Hydroxytyrosol (3,4-dihydroxyphenylethanol), which is produced when 3,4-dHPAA is reduced by yeast oxidoreductases, increased with promoter strength, indicating that MAO was successfully generating 3,4-dHPAA. Crucially, successful restoration of (*S*)-reticuline synthesis in a promoter-dependent manner also confirmed that 3,4-dHPAA can not only be produced but also condensed with dopamine in yeast to produce (*S*)-norlaudanosoline, which can then be methylated by 6OMT, CNMT, and 4́OMT to form (*S*)-reticuline (Figure 1B).

**Figure 3:**
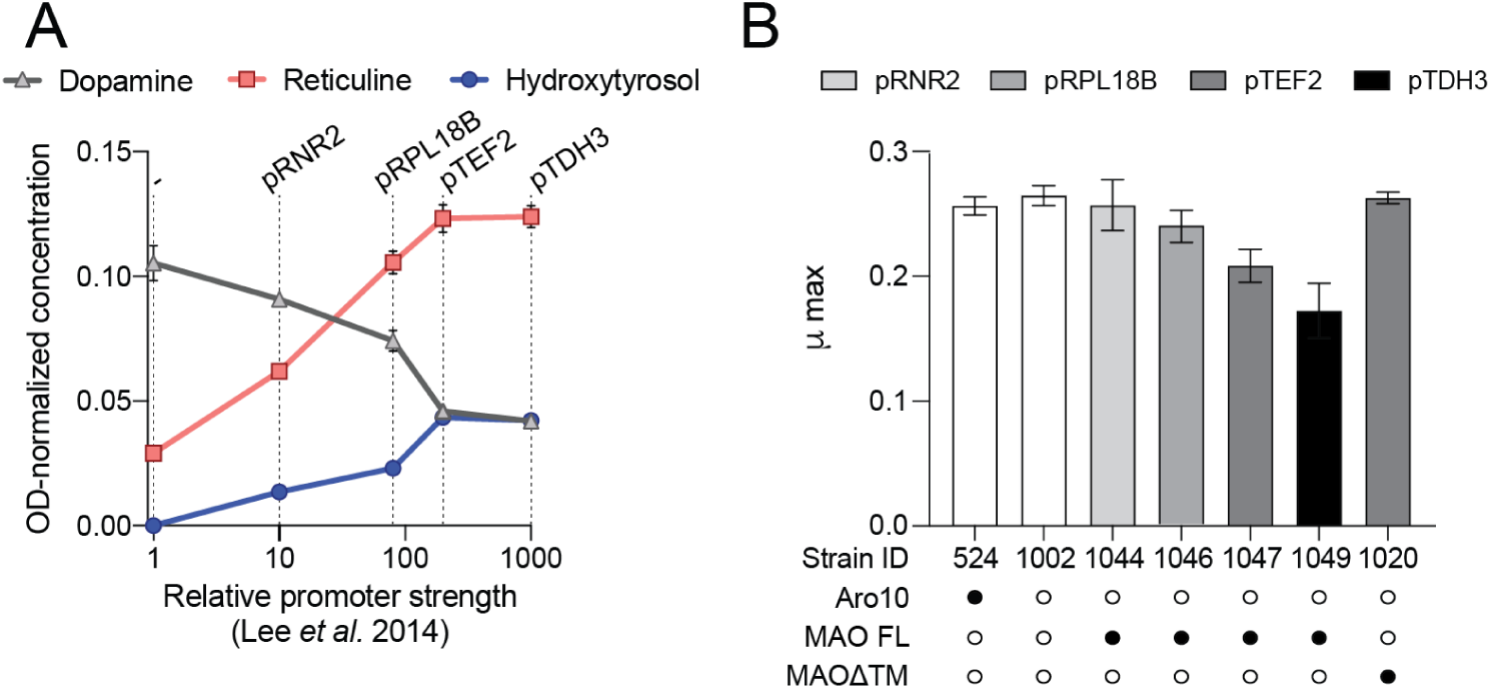
Promoter titration of *MAO* in an *ARO10* knockout background. Strain LN1002 lacking Aro10 was used to express *MAO* under the control of *pRNR2*, *pRPL18B*, *pTEF2,* or *pTDH3*. (A) Cultures were grown in rich media in deep well plates and then analyzed for the metabolite profile and final OD_600_. Metabolite profile of strains is presented as g/L normalized to OD_600_. Error bars refer to mean and standard deviation of *n* = 2 biological replicates. (B) Cultures were grown in minimal media in microtiter plates in a plate reader, and maximum specific growth rate was obtained from the growth curves. Promoter strength driving MAO expression is indicated by gray shade. Abbreviations: MAO FL, full-length MAO; MAOΔTM, MAO with transmembrane helix deleted. Symbols: ○ no enzyme; • one enzyme copy. Error bars refer to mean and standard deviation of *n* = 3 biological replicates.

Growth rate assays performed in parallel with metabolite analyses revealed that strains’ maximum specific growth rate (μ_max_) decreased with increased *MAO* expression (Figure 3B). In humans, MAO harbors a 30 amino acid C-terminal transmembrane helix anchored in the outer mitochondrial membrane.^29,30^ We investigated whether mitochondrial localization of MAO affected the growth rate by removing the transmembrane helix and expressing this variant (MAOΔTM) in yeast under the control of the *TEF2* promoter (strain LN1020). Removal of the transmembrane helix restored μ_max_ to the level observed in strains not expressing MAO (Figure 3B), but it did not impact MAO localization (Supplemental Figure 1). We also observed that the activity of MAOΔTM was lower than that of its full-length counterpart (Figure 2A). Further, we conclude that the improved growth rate of cells expressing *MAOΔTM* was due to reduced enzymatic activity and not due to altered localization of the enzyme. This corroborates a prior report in which MAOΔTM was demonstrated to maintain localization at mitochondria while having difficulty forming disulfide bridges essential for full enzymatic activity.^31^

Synthesis of 3,4-dHPAA from dopamine also produces a molar equivalent of hydrogen peroxide, which may impose a burden on the cell and reduce the μ_max_. To mitigate these effects, we attempted to target MAO to the peroxisome using the peroxisomal targeting tag ePTS1 (ref.^32^) (LN1015; MAOΔTM-p). However, there was no evidence that MAO was localized to the peroxisome, with or without removal of the transmembrane helix (Figures 1 and 3). Further studies are required to better understand how to manipulate MAO localization without also perturbing enzyme activity.

### *MAO* Driven by *pTEF2* Limits Off-Target Tetrahydroisoquinoline Synthesis in Fed-Batch Fermentation

A key challenge of BIA production in *S. cerevisiae* is its propensity to transform precursor aldehydes into fusel alcohols or acids.^7^ Aldehydes may be oxidized or reduced by oxidoreductases depending on the redox environment of the cell.^19^ In our prior study, significant residual 4-HPAA redox activity remained in strain LP507, even with the deletion of seven oxidoreductases. Oxidation of 4-HPAA into 4-hydroxyphenylacetic acid (4-HPAC) was especially persistent. In that study, we circumvented this issue by utilizing a fed-batch protocol that promoted periodic production and reuptake of ethanol—a series of sugar pulses controlled through off-gas analysis—intended to maintain an environment where yeast has limited capacity to oxidize 4-HPAA.^33^ A downside of this protocol is carbon loss in the repeated phases of fermentation growth and ethanol production. Moreover, utilizing a simple, sugar-limited feeding profile would be a preferred solution for BIA synthesis in industrial settings. Thus, we sought to establish an exponential fed-batch regime in *MAO*-expressing strains, using the experiment with strain LP524 as a baseline for comparison.^7^

Compared to LP524, strain LN1047 expressing *MAO* from the *TEF2* promoter produced more (*S*)-reticuline (3.0 vs 4.0 g/L; Figure 4B). Crucially, strain LN1047 made fewer 4́-unsubstituted benzyl-THIQs (5% vs 33% of the total peak area; Figure 2B). In total, (*S*)-reticuline comprised 80% of the total THIQs by peak area, compared to 49% for LP524. These initial results demonstrated that BIA synthesis through 3,4-dHPAA successfully reduced the production of off-target condensation products. However, the presence of 3.2 g/L hydroxytyrosol, 0.16 g/L 3,4-dHPAC, and 0.44 g/L dopamine indicated a need for additional strain optimization.

**Figure 4:**
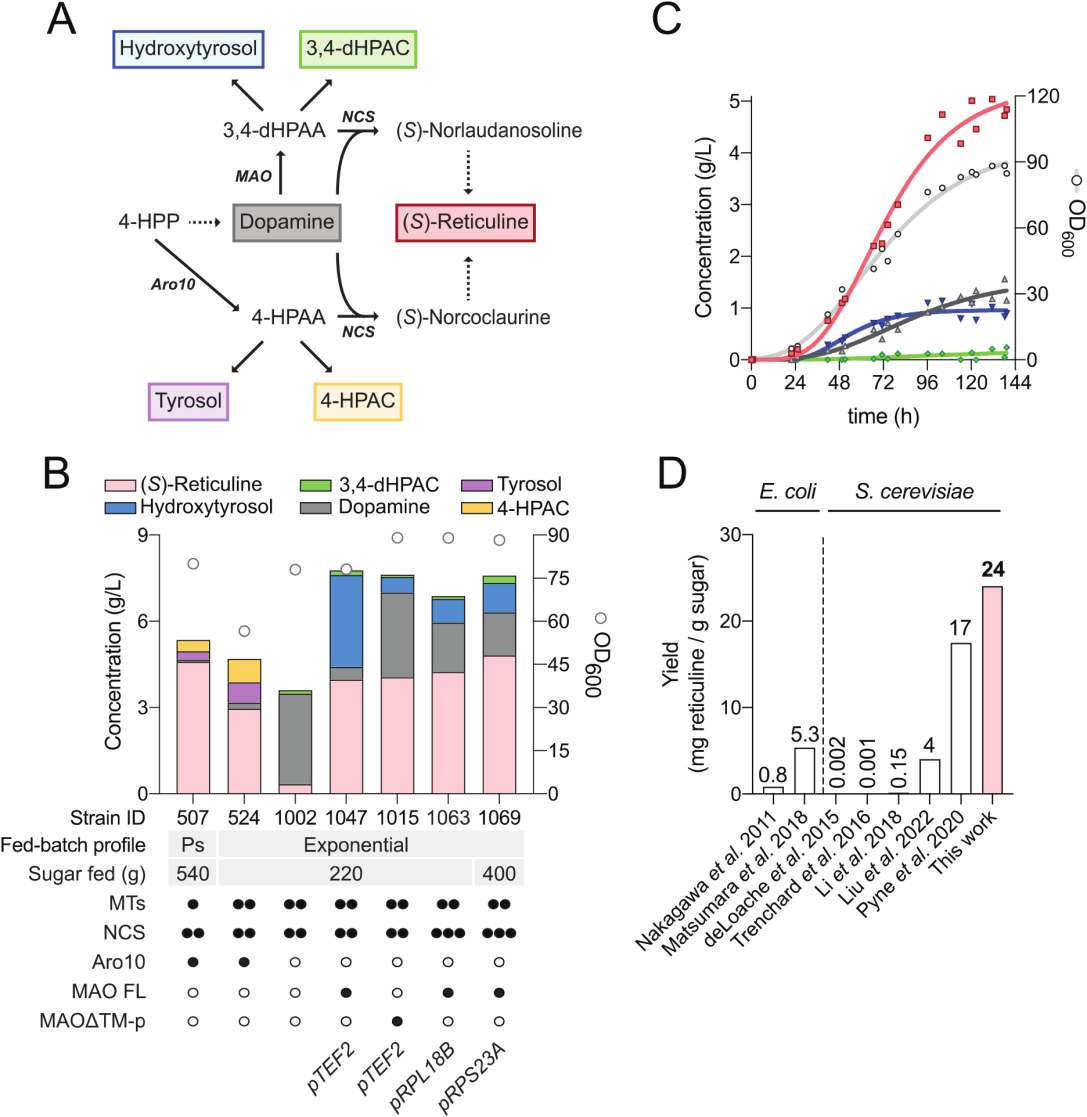
Optimization of (*S*)-reticuline production in fed-batch fermentation. (A) Pathways for fusel acid and alcohol production in strains synthesizing 4-HPAA and 3,4-dHPAA. Dashed arrows refer to multiple enzymatic reactions. (B) End-point analysis of quantifiable metabolites of BIA-producing strains grown in fed-batch fermentation. Strains were grown in the batch phase until sugar was exhausted, then one of two sugar feeding fed-batch profiles was used—pulsed (Ps) or exponential. Pulsed fed-batch media contained 500 g sucrose/L, and exponential fed-batch media contained 360 g sucrose/L. Bar graph colors correspond to metabolites in Panel A. White circles indicate OD_600_ values plotted on the secondary axis. (C) Time course of fed-batch fermentation with LN1069, with *MAO* expression under control of *pRPS23A*. Two replicates are plotted with a best-fit curve included. Line graph colors correspond to metabolites in Panel A. (D) *De novo* reticuline yields reported in the literature expressed as milligrams of reticuline/g of sugar. Compound abbreviations: 4-HPP, 4-hydroxyphenylpyruvate; 4-HPAC, 4-hydroxyphenylacetate; 3,4-dHPAA, 3,4-dihydroxyphenylacetaldehyde; 3,4-dHPAC, 3,4-dihydroxyphenylacetate. Enzyme abbreviations: MTs, methyltransferases; NCS, norcoclaurine synthase. Symbols: ○ no enzyme; • one enzyme copy; •• two copies; ••• three copies.

### Balancing Expression of Pathway Branch Point Enzymes Enhances Norlaudanosoline Synthesis

Rerouting (*S*)-reticuline synthesis using MAO improved the selectivity of THIQ synthesis (Figure 2A and B), but the accumulation of dopamine and the 3,4-dHPAA-derived hydroxytyrosol posed a new problem (Figure 4B). A delicate balance must be achieved in the synthesis of dopamine, 3,4-dHPAA, and (*S*)-norlaudanosoline (Figure 4A): MAO activity for the conversion of dopamine to 3,4-dHPAA must not be too high or too low, while NCS activity must be sufficient to condense dopamine and 3,4-dHPAA before the former exits the cell or scavenging oxidoreductases convert the latter to hydroxytyrosol and 3,4-dHPAC. When *MAO* was expressed from the *TEF2* promoter, the concentration of hydroxytyrosol was 6-fold higher than that of dopamine in fed-batch fermentation, suggesting excessive MAO activity. Thus, two options were explored to alter the ratio of dopamine and 3,4-dHPAA: using the MAO variant MAOΔTM-p with reduced activity compared with wild-type MAO and decreasing the strength of the promoter driving *MAO* expression.

In fed-batch fermentation, *MAOΔTM-p* under the control of the *TEF2* promoter (strain LN1015) resulted in the synthesis of 4 g/L (*S*)-reticuline, in this instance with the additional accumulation of 3 g/L dopamine and 0.6 g/L hydroxytyrosol (Figure 4B), pointing to insufficient activity of MAO. Additionally, the continued accumulation of both dopamine and hydroxytyrosol in fermentations with strains LN1047 and LN1015 suggested that NCS activity may not be sufficient for the efficient condensation of dopamine and 3,4-dHPAA (Figure 4B). *NCS* expression negatively impacts strain fitness, which can be alleviated by peroxisomal sequestration.^32^ Thus, an extra copy of peroxisomally targeted NCS was introduced to strains moving forward.

To address precursor imbalance, we probed weaker promoters driving full-length *MAO* expression. With *MAO* under the control of the *RPL18B* promoter, strain LN1063 produced 4.25 g/L (*S*)-reticuline, 1.5 g/L dopamine, and 0.5 g/L hydroxytyrosol (Figure 4B). Although this resulted in a slight improvement in (*S*)-reticuline titer, dopamine concentration was still three times higher than that of hydroxytyrosol, indicating that optimal MAO activity in this strain may require a promoter with strength between *pRPL18B* and *pTEF2*.

The yeast MoClo collection of characterized promoters does not include any with strength intermediate to *pRPL18B* and *pTEF2* (ref.^28^ ). A 2010 report from Canelas et al.^34^ contains RNASeq data of two common yeast strains, CEN.PK and S288C, grown in both shake-flask and sugar-limited chemostat conditions. We searched these data for promoters whose strength was between those of *pRPL18B* and *pTEF2* in both CEN.PK and S288C (the strains in this study are based on the latter) under both shake-flask and sugar-limited conditions. Three promoters, *pRPL39*, *pHTB1*, and *pRPS23A*, were selected. We confirmed that these promoters were indeed intermediate to *pRPL18B* and *pTEF2*, as determined by dopamine, hydroxytyrosol, and (*S*)-reticuline abundance in a 96-well plate format (Supplemental Figure 2B). From this set, strain LN1069, expressing full-length *MAO* from *pRPS23A* as well as a second copy of peroxisomally targeted *NCS*, was selected for further characterization in a 3 L bioreactor (Figure 4C).

In fed-batch cultivation, strain LN1069 synthesized 4.8 g/L (*S*)-reticuline, with an additional 1.5 g/L dopamine and 1.0 g/L hydroxytyrosol (Figure 4B and C). In this experiment, we increased the total sucrose fed to 400 g, corresponding to an overall yield of 24 mg (*S*)-reticuline/g sucrose (Figure 4D). By mass spectrometry, (*S*)-reticuline comprised 83% of THIQs with another 12% attributable to benzyl-THIQs and 5% to 4́-unsubstituted benzyl-THIQs (Figure 2B).

### *De Novo* Synthesis of Dihydrosanguinarine via (*S*)-Norlaudanosoline

Previously, we introduced a pathway into yeast to convert supplemented (*S*)-norlaudanosoline to dihydrosanguinarine.^35^ In that work, we identified a pathway bottleneck between (*S*)-scoulerine and (*S*)-stylopine synthesis, which was resolved in a follow-up report.^36^ In the present work, all six enzymes were introduced into a yeast strain synthesizing (*S*)-reticuline *de novo* via (*S*)-norlaudanosoline (strain LN1015) (Figure 5A).

**Figure 5:**
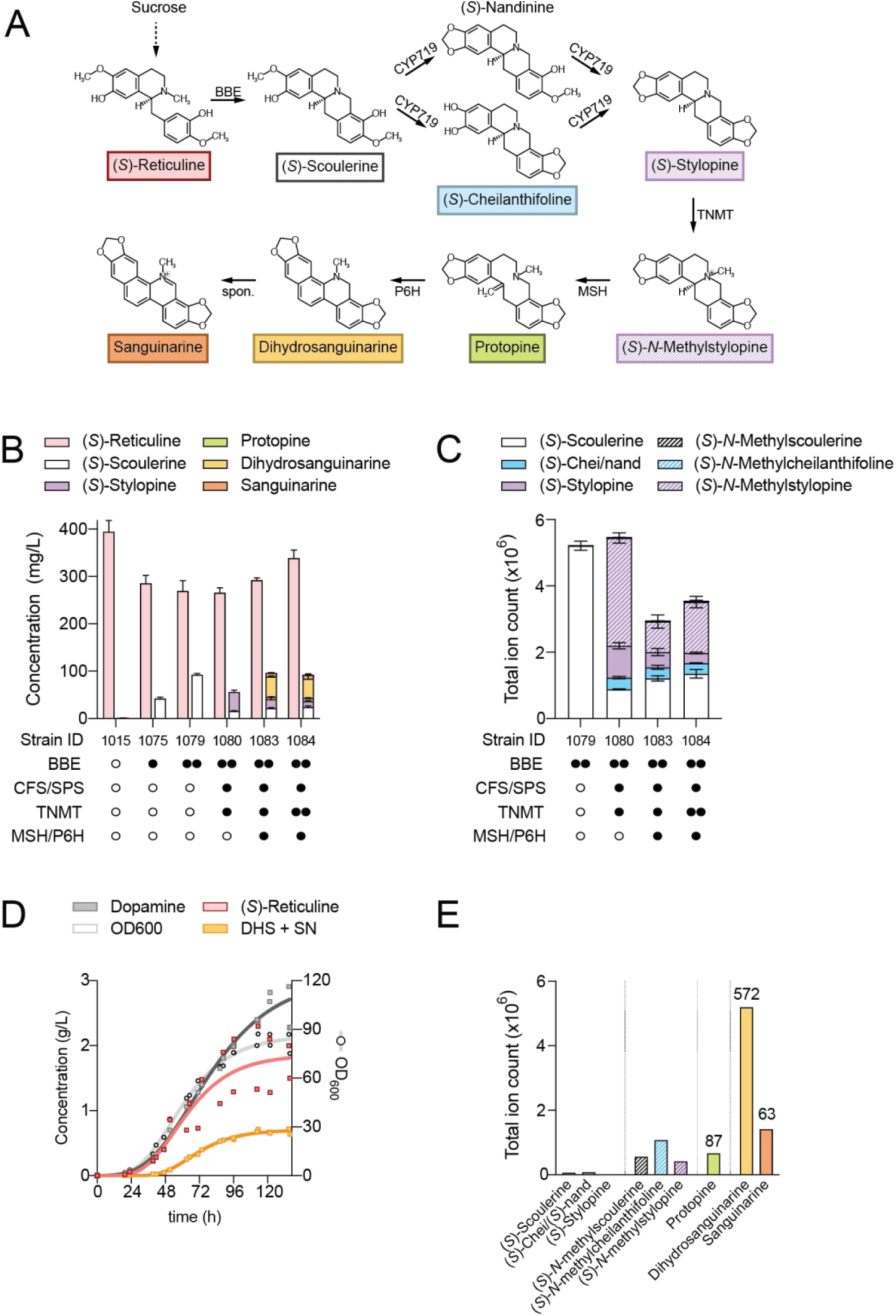
*De novo* dihydrosanguinarine synthesis in yeast. (A) Biosynthesis of dihydrosanguinarine from (*S*)-reticuline. (B) Stepwise construction of a *de novo* dihydrosanguinarine strain. Pathway enzymes were integrated into LN1015, a strain synthesizing (*S*)-reticuline via (*S*)-norlaudanosoline. Metabolites were extracted and, where possible, quantified by LC-MS. Error bars refer to mean and standard deviation of *n* = 3 biological replicates. Unidirectional error bars are shown to avoid overlap across metabolites. (C) Metabolite profile of intermediates between (*S*)-scoulerine and (*S*)-*N*-methylstylopine. Error bars refer to mean and standard deviation of *n* = 3 biological replicates. (D) Fed-batch fermentation of LN1084, a strain synthesizing dihydrosanguinarine *de novo*. Samples were regularly collected for OD_600_ and metabolite analysis. Two replicates are plotted with a best fit line. (E) Metabolite profile of target and intermediate compounds at the final time point of a representative fed-batch fermentation in panel (D). Concentrations of quantifiable metabolites are indicated in mg/L. Abbreviations: BBE, berberine bridge enzyme; CFS, (*S*)-cheilanthifoline synthase; SPS, (*S*)-stylopine synthase; TNMT, (*S*)-tetrahydroprotoberberine *N*-methyltransferase; MSH, (*S*)-*N*-methylstylopine hydroxylase; P6H, protopine 6-hydroxylase; DHS, dihydrosanguinarine; SN, sanguinarine. Symbols: ○ no enzyme; • one enzyme copy; •• two copies.

Introduction of one copy of the gene encoding the berberine bridge enzyme (*BBE*) to strain LN1015 resulted in 15% conversion of (*S*)-reticuline to (*S*)-scoulerine in deep-well plates (Figure 5B, strain LN1075). A second copy of *BBE* improved conversion to 25% (LN1079). The next three pathway enzymes, (*S*)-cheilanthifoline synthase (CFS), (*S*)-stylopine synthase (SPS), and (*S*)-tetrahydroprotoberberine *N*-methyltransferase (TNMT) were introduced into strain LN1079, generating strain LN1080 (Figure 5B). In our 2016 report, these three enzymes converted 100% of supplemented (*S*)-scoulerine to (*S*)-*N*-methylstylopine.^36^ Here, conversion was incomplete, as 15 mg/L of (*S*)-scoulerine was detected, along with residual (*S*)-cheilanthifoline. A minor bottleneck at (*S*)-stylopine methylation was also observed. Finally, the two cytochromes P450 (*S*)-*N*-methylstylopine hydroxylase (MSH) and (*S*)-*N*-methylstylopine hydroxylase (P6H) were introduced to LN1080, generating a strain of yeast producing dihydrosanguinarine *de novo* from sucrose (Figure 5B, LN1083). In deep-well plates, LN1083 synthesized 40 mg/L of dihydrosanguinarine. Additionally, 7 mg/L of sanguinarine was produced, presumably through the spontaneous oxidation of dihydrosanguinarine. The impact of a second copy of *TNMT* was also probed (strain LN1084). While there was no improvement in dihydrosanguinarine titers, we observed a decrease in (*S*)-stylopine and an increase in (*S*)-*N*-methylstylopine, indicating that the extra copy of *TNMT* was effective (Figure 5C).

Growth of strain LN1084 in sugar-limited fed-batch conditions resulted in the synthesis of 572 mg/L dihydrosanguinarine and an additional 63 mg/L sanguinarine for a combined output of 635 mg/L (Figure 5D). Additionally, 2.2 g/L of (*S*)-reticuline and 87 mg/L of protopine accumulated in the broth. BBE remained the rate-limiting step in flux through the dihydrosanguinarine pathway in fed-batch conditions, in accordance with experiments in deep-well plate format. (*S*)-Scoulerine, (*S*)-cheilanthifoline, and (*S*)-stylopine were almost undetectable, whereas the three *N*-methylated equivalents accumulated (Figure 5E).

### *De Novo*Synthesis of Side Products Upon Introduction of Dihydrosanguinarine Synthesis

Several novel peaks were observed upon coexpression of enzymes to convert (*S*)-reticuline to dihydrosanguinarine. Two distinct peaks with exact [M + H]^+^ = 356.1490 (C_20_H_21_NO_5_) and similar retention times were identified (Figure 6A). The chemical formula and MS^2^ profile suggested that these compounds are closely related to protopine and consistent with hunnemanine and izmirine (Figure 6B). These compounds are proposed to be derived from the off-target activity of MSH on (*S*)-*N*-methylcheilanthifoline and (*S*)-*N*-methylnandinine rather than on (*S*)-*N*-methylstylopine (Figure 6C). Assuming equal ionization efficiency to protopine, these peaks could represent 30 mg/L of lost carbon.

**Figure 6:**
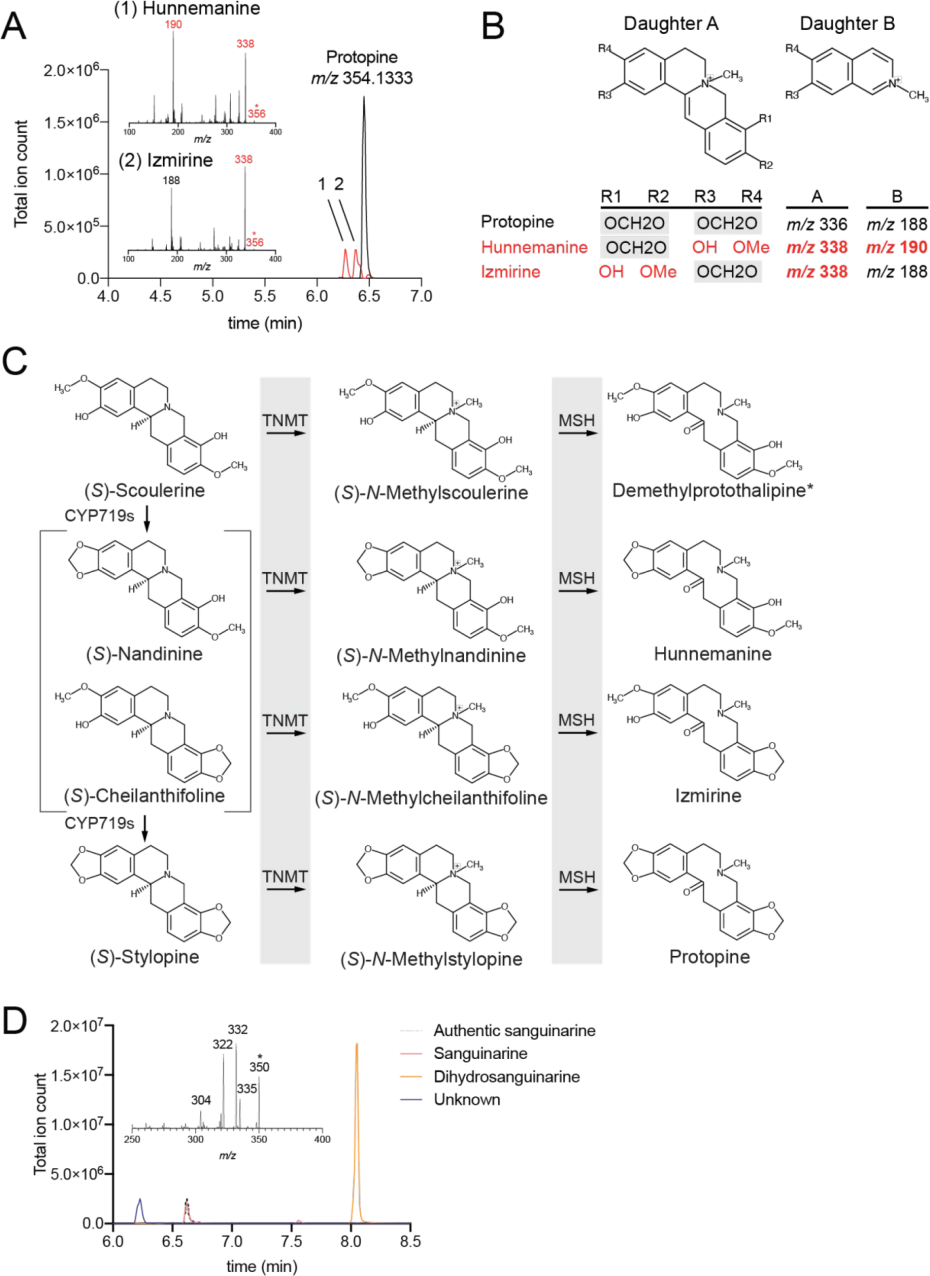
Unidentified metabolites in dihydrosanguinarine fermentation samples of strain LN1084. Broth from the fed-batch fermentation of strain LN1084, synthesizing *de novo* dihydrosanguinarine, was extracted and analyzed by HPLC-FT-MS/MS. (A) Two unknown peaks with exact *m*/*z* [M + H]^+^ = 356.1490 were observed; chromatograms and MS^2^ fragmentation patterns are displayed, with MS^2^ daughter ions differing by 2 *m*/*z* from protopine highlighted in red. (B) Two MS^2^ fragmentation ions characteristic of protopines, which can be used to distinguish unknowns 1 and 2. (C) Synthesis of hunnemanine and izmirine is proposed to proceed through activity of the enzyme MSH on the *N*-methylated pathway side-products *N*-methylnandinine and *N*-methylcheilanthifoline, respectively. (D) A peak with exact *m*/*z* [M + H]^+^ = 350.1025 was observed; chromatograms and MS^2^ fragmentation patterns are displayed.

We also detected an additional unknown compound with [M + H]^+^ = 350.1025 (C_20_H_17_NO_5_) (Figure 6D). The compound is likely a benzophenanthridine like sanguinarine, or a dihydrobenzophenanthridine like dihydrosanguinarine, displaying characteristic losses of [M-Me]^+^ (15 *m*/*z*) and [product ion + H–CO]^+^ (28 *m*/*z*) as well as a fragment corresponding to the exact mass of sanguinarine (*m*/*z* = 322).^37^ Its chemical formula corresponds to a hydroxydihydrosanguinarine, yet its MS^2^ profile does not match that of 10-hydroxydihydrosanguinarine.^38^ Alternatively, 6-hydroxydihydrosanguinarine (also known as sanguinarine pseudobase) is a naturally occurring form of sanguinarine that exists in equilibrium with sanguinarine and sanguinarine dimer at physiological pH.^39^ However, an authentic MS^2^ profile of the sanguinarine pseudobase could not be obtained for validation of this hypothesis. Sanguinarine pseudobase is not observed in an authentic sanguinarine standard (Figure 6C), but the standard was not held at physiological pH. Assuming an ionization efficiency equal to that of sanguinarine, this compound could represent an additional 100 mg/L of material derived from (*S*)-reticuline.

## Discussion

In 2020, we reported the first gram-per-liter-scale synthesis of the BIA (*S*)-reticuline in a microbial host.^7^ However, the pathway suffered from excessive accumulation of side-products formed through the combined broad activities of the yeast Ehrlich pathway enzyme Aro10 and plant NCS variants. Here, we design and employ an alternative pathway by rerouting the (*S*)-reticuline synthesis through (*S*)-norlaudanosoline using MAO (Figure 1B). This MAO-dependent route results in an improved (*S*)-reticuline yield (24 vs 17 mg/g glucose), titer (4.8 vs 4.6 g/L), and selectivity (83 vs 42%) (strain LN1069) relative to our previous Ehrlich-dependent pathway (strain LP524).

Enzyme promiscuity was a frequent theme throughout this work. Yeast could catabolize the non-native aldehyde 3,4-dHPAA, yielding either hydroxytyrosol or 3,4-dHPAC based on culture conditions. 4́-Unsubstituted THIQ synthesis required the concerted, promiscuous activity of Aro10, NCS, and 6OMT and CNMT methyltransferases (Figure 1). Further, upon the introduction of *de novo* dihydrosanguinarine synthesis, multiple side products were discovered, which required off-target activity of both TNMT and MSH (Figure 5E). Although the synthesis of 4́-unsubstituted THIQs was largely resolved in this work, additional challenges remain.

One general strategy for addressing enzyme promiscuity is to ensure that the heterologous pathway of interest is functioning linearly and with minimal bottlenecks. This is traditionally achieved using standard approaches, such as copy number titration or identification of orthologous enzymes with superior specificity or activity in yeast. Heterologous enzymes may need modifications to function optimally in a yeast host, as activity of the NCS variant used here was previously improved through deletion of an N-terminal signal peptide.^7^ Other options to limit the impact of promiscuity include enzyme colocalization through scaffolding, fusion, or compartmentalization into natural or synthetic organelles.^40,41^

A complementary strategy is to ensure that the native metabolism of the yeast host is fine-tuned to be compatible with the heterologous pathway as much as possible. For instance, the methyltransferases converting (*S*)-norlaudanosoline to (*S*)-reticuline depend on *S*-adenosylmethionine (SAM). It may be beneficial to introduce SAM cofactor engineering^42^ to facilitate the conversion of (*S*)-norlaudanosoline to the triple-methylated (*S*)-reticuline, especially in gram-per-liter-scale syntheses. This could reduce the accumulation of benzyl-THIQ pathway intermediates, which comprised 12% of the total THIQs in the final strain LN1069 (Figure 2B).

Paradoxically, we successfully leveraged enzyme promiscuity to improve pathway specificity; rerouting (*S*)-reticuline synthesis through 3,4-dHPAA required the enzymes NCS, 6OMT, and CNMT to act on noncanonical substrates (Figure 1B). By virtue of this, strain LN1069 produced just 5% of off-target 4́-unsubstituted benzyl-THIQs, down from 33% in LP524 (Figure 2B). However, strains producing (*S*)-reticuline through (*S*)-norcoclaurine generated relatively little 4-HPAA-derived fusel alcohol (tyrosol) and acid (4-HPAC), while strains producing (*S*)-norlaudanosoline exhibited significant accumulation of fusel products (hydroxytyrosol and 3,4-dHPAC) (Figure 2B, Figure 4A and B). This finding suggests that problems with off-target THIQ and precursor diversion were effectively reversed in the two lineages. Somewhat surprisingly, the major degradation product of 3,4-dHPAA in strain LN1069 was hydroxytyrosol, a non-native fusel alcohol (Figure 4B), in spite of the deletion of seven oxidoreductases (*ari1*Δ *adh6*Δ *ypr1*Δ *ydr541c*Δ *aad3*Δ *hfd1*Δ *gre2*Δ) which dramatically reduced the accumulation of the closely related tyrosol in LP524 (ref.^7^ ). This illustrates the redundancy and efficacy of yeast’s Ehrlich pathway.^18,19^ Additional deletions may be required to ensure efficient function of this key branch point and key candidates include Ald4, Gcy1, Gre3, and Ydl124w.^18,43^ However, some enzymes demonstrated to act on aromatic aldehydes, such as Ald4 (ref.^7^ ), cannot be easily removed from an industrially viable host.^18^

In addition to the MAO-dependent pathway constructed in this study, an alternative route to 3,4-dHPAA and norlaudanosoline has been demonstrated in yeast using the Ehrlich pathway. By analogy to the Ehrlich-mediated conversion of l-tyrosine to 4-HPAA, deamination (via Aro8/Aro9) and decarboxylation (Aro10) of l-DOPA yields 3,4-dHPAA. Accordingly, growing dopamine-producing NCS strains on l-DOPA as a sole nitrogen source confirmed the production of norlaudanosoline.^7^ However, growth of the same engineered strain on urea as nitrogen source failed to yield norlaudanosoline despite the presence of l-DOPA, incidating that conversion of l-DOPA to dopamine by l-DOPA decarboxylase (DODC) outcompeted the Ehrlich pathway for l-DOPA availability. Further, our present study inactivated the Ehrlich pathway (*aro10*Δ), thus eliminating this alternative route as a possible source of 3,4-dHPAA and norlaudanosoline.

Strains expressing *MAO* also accumulated more unreacted dopamine than strains overexpressing *ARO10* in fed-batch fermentations (Figure 4B). Recapturing the carbon lost to fusel products and dopamine could result in as much as a 50% increase in the (*S*)-reticuline titer and productivity metrics in these strains. However, the pathway branch points at dopamine, 3,4-dHPAA, and (*S*)-norlaudanosoline (Figure 4A) must be approached delicately. While increasing *NCS* expression through increasing gene copy number may prove useful for overcoming precursor accumulation, the burden of high NCS load may limit the utility of this approach.^32^ Alternatively, entry into the aromatic amino acid pathway could be dynamically controlled using a transcriptional biosensor specific to a pathway side product such as hydroxytyrosol.^44^ Dynamic control at this critical branch point could also be achieved by employing a dopamine-sensing promoter such that conversion to 3,4-dHPAA is achieved in a concentration-dependent manner to prevent dopamine accumulation. We noticed dopamine concentration continued to rise toward the end of fermentation while those of hydroxytyrosol and (*S*)-reticuline largely plateaued (Figure 4C). This could be attributed to a decrease in MAO activity or changes in expression levels of other native and non-native enzymes. A broader scan of promoters for *MAO* expression may identify a candidate that is more appropriate for maintaining consistent BIA production independent of growth rate.^34,45^

Biomass yield also began to plateau toward the end of fed-batch fermentations of BIA-synthesizing strains, whether they produced (*S*)-reticuline via 3,4-HPAA (Figure 4C) or 4-HPAA (Supplemental Figure 4). Growth plateaued largely in accordance with (*S*)-reticuline synthesis, suggesting that BIA production is at least partially growth-coupled. It is likely that the engineered strain background has multiple sources of stress, the effects of which are exacerbated over the course of a fermentation. LP524, producing (*S*)-reticuline through 4-HPAA, reached a lower biomass density than the *aro10*Δ*L*N1002 strain and the final OD remained higher even when MAO was introduced and (*S*)-reticuline synthesis was restored (Figure 4B; strains 1047, 1015, 1063, and 1069). Thus, MAO strains experience reduced metabolite toxicity, redox imbalance, or stress response relative to the alternative Ehrlich route, which is ostensibly due to the expression of *ARO10* and the presence of 4́-unsubstituted THIQs or excess fusel aldehyde accumulation. Higher *MAO* expression level was accompanied by a reduction in maximum specific growth rate in 96-well plates. Redirection of MAO to the peroxisome was unsuccessful (Supplemental Figures 1 and 3), but additional approaches to mitigating hydrogen peroxide stress, such as catalase overexpression,^46^ could be explored. Deleterious effects of elevated intracellular BIAs cannot be ruled out, in which case engineering BIA export may alleviate some growth inhibition.^47^

The BIA sanguinarine is a primary active ingredient in Sangrovit, a probiotic used in animal husbandry. To demonstrate the commercial potential of our yeast BIA platform, an (*S*)-reticuline producing strain (LN1015) was modified to produce dihydrosanguinarine (LN1084) directly from sugar. In fed-batch fermentation, we produced 635 mg/L dihydrosanguinarine and its oxidized derivative sanguinarine from sucrose. This represents the highest titer of those BIAs produced in microbes by a factor of 40 (ref.^25^) and is a sufficient quantity to serve as a probiotic for approximately one ton of fish feed.^48,49^ Our work also demonstrates *de novo* synthesis of putative protopine metabolites consistent with hunnemanine and izmirine. These compounds were synthesized due to a minor bottleneck at the level of (*S*)-stylopine synthesis. While this bottleneck was previously resolved,^36^ it may have reappeared due to the improved efficiency of *de novo* (*S*)-scoulerine synthesis herein. More than two mM of (*S*)-scoulerine-derived BIAs were produced in this study using strain LN1084 in fed-batch fermentation (Figure 5E) vs 5 μM of exogenous (*S*)-scoulerine supplementation in previous work. Conversion of (*S*)-reticuline to (*S*)-scoulerine was also incomplete, highlighting a previously described major bottleneck at BBE.^25^ BBE has been shown to be poorly soluble in *E. coli*, which was ameliorated by generating an N-terminal maltose-binding fusion protein, resulting in an 80-fold improvement in BBE activity *in vivo*.^50^ This fusion protein strategy may also prove beneficial in our strains, potentially enabling commercially viable production of (*S*)-scoulerine-derived BIAs including protoberberines, protopines, benzophenanthridines, and phthalideisoquinolines in the future.

In summary, we engineered yeast to synthesize (*S*)-reticuline via (*S*)-norlaudanosoline, enabling improved productivity metrics compared to the previously reported (*S*)-norcoclaurine route. We also extended the heterologous pathway to dihydrosanguinarine, resulting in the highest titer of commercial BIA in a microbial host. We highlight the importance of monitoring both on- and off-pathway metabolites, challenges with reducing shunting of pathway intermediates and balancing enzyme expression levels at key branch points in yeast THIQ biosynthesis.

## Materials and Methods

### Yeast and *E. coli* Growth Conditions

*E. coli* was grown in liquid Luria Broth (10 g/L peptone, 5 g/L yeast extract, 10 g/L sodium chloride, LB; Fisher Bioreagents) at 37 °C with shaking at 200 rpm. *E. coli* transformations were selected on solid LB with 2% agar with antibiotics supplied as necessary.

For genetic manipulation, yeast was grown in liquid yeast peptone dextrose (20 g/L peptone, 20 g/L dextrose, 10 g/L yeast extract, YPD; Sigma-Aldrich) at 30 °C while being shaken at 200 rpm. Yeast transformations with Cas9-containing plasmids were selected on solid YPD with 2% agar, 200 μg/mL G418, and 200 μg/mL hygromycin. For assessment of BIA synthesis in 96-well plate format, yeast was grown in 2x synthetic complete media (2x SC: 13.6 g/L Difco Yeast Nitrogen Base (YNB), 3.84 g/L yeast synthetic dropout medium supplements without histidine (Millipore-Sigma), 152 mg/L histidine, 40 g/L sucrose) with shaking at 400 rpm in 96-well 2 mL deep-well plates overnight, followed by a 1:50 back dilution into fresh 2x SC for 3 days. For assessment of yeast growth in a 96-well plate format, strains were grown in 2x SC overnight with shaking at 400 rpm, followed by a 1:100 back dilution into YNB with 20 g/L sucrose supplemented with 76 mg/L methionine and 76 mg/L histidine. Prior to growth in the fermenter, strains were transformed with a plasmid complementing methionine and histidine auxotrophies and selected on solid 1x SC media lacking histidine (SC-His).

### Strain Construction

Gene knockouts and genomic integrations were introduced into yeast via CRISPR-directed homologous recombination. A plasmid harboring Cas9 and an empty guide RNA transcription cassette was linearized by *Not*I + *Bsa*I double digestion (New England Biolabs) and used to transform yeast together with a linear piece of DNA containing the guide RNA sequence flanked on either side by homology to the plasmid, which resulted in an *in vivo* plasmid assembly. Linear DNA containing the guide was generated by PCR. Guide RNAs for gene knockout were selected based on a combined score from the online tools Yeast CRISPRi^51^ and CCTOP.^52^ Gene knockouts were generated through cotransformation of a linear fragment containing 40 bp homology to either side of the gene of interest. Gene integrations were targeted to genomic regions previously identified to promote high-level *GFP* expression.^53,54^ Gene integrations were introduced either as precloned promoter-gene-terminator cassettes or as individual promoters, genes, and terminators containing 40 bp of overlap between parts. Integrations were targeted to a region of interest in *trans* through cotransformation of ∼ 600 bp regions of homology to the genome, with 40 bp of homology to common linker sequences present at the 5′ and 3′ ends of gene cassettes. All transformations were performed using a standard lithium acetate/salmon sperm heat shock protocol. Yeast strains were cured of Cas9-containing plasmids between rounds of transformations by substreaking on solid YPD.

The vector pGC1899, harboring expression cassettes for *SdiCFS*, *NdoSPS*, and *PsTNMT*, was constructed via a Golden Gate assembly. Type IIS enzymes were purchased from Thermo Fisher Scientific, T7 ligase and T4 ligase buffer were purchased from New England Biolabs. Golden Gate reactions were performed as described in the Yeast Toolkit (YTK) system.^28^ Assemblies containing *SdiCFS* were performed using “end on ligation”, due to the presence of an internal *Bsa*I site.

### Growth Curves and Determination of Maximum Growth Rate

Yeast was grown in a Tecan Sunrise plate reader. Plates were wrapped with Parafilm to prevent evaporation. Growth measurements (OD_595_) were taken every 5 min for 48 h. Following background subtraction, values were normalized to starting OD, ln-transformed, and smoothed across a 20 min interval. The slope of the curves across rolling 1-h windows was determined, and the maximum slope was considered μ_max_.

### Fed-Batch Fermentation

Fed-batch fermentations were performed in an Applikon 3L BioBundle fermenters. pH was maintained at 4.5 using 4N NaOH, temperature was kept at 30 °C. Air flow was set to 1 L/min, and dissolved oxygen was controlled at 35% air saturation by automatic adjustment of stirring rate. Off-gas composition (partial pressure of O_2_ and CO_2_) was measured with a Tandem Multiplex gas analyzer. Precultures were grown at 30 °C for 36 h in 50 mL of SC-His medium with shaking at 200 rpm. Cells were centrifuged for 10 min at 4000 *g*, washed in 0.9% NaCl, and suspended in 50 mL 0.9% NaCl prior to inoculation in 950 mL of batch medium (initial OD_600_∼0.2). Following exhaustion of sugar as indicated by off-gas analysis, fed-batch phase was triggered with an initial feeding rate of 0.60 g/h sucrose and increased exponentially at a dilution rate of 0.025 h^–1^. Batch medium (per liter): 40 g of sucrose, 6 g of (NH_4_)_2_SO_4_, 2.5 g/L KH_2_PO_4_, 1 g of MgSO_4_·7H_2_O, 5 mL of vitamin stock, and 5 mL of trace element stock. Feeding medium (per liter): 360 g of sucrose, 60 g of (NH_4_)_2_SO_4_, 15 g of KH_2_PO_4_, 6 g of MgSO_4_·7H_2_O, 15 mL of vitamin stock, and 15 mL of trace element stock per liter. Vitamin stock (per liter): 2,500 mg myo-inositol, 100 mg calcium pantothenate, 100 mg thiamine hydrochloride, 100 mg pyridoxine, 100 mg nicotinic acid, 20 mg *p*-aminobenzoic acid, 5 mg biotin, and 5 mg folic acid. Trace element stock (per liter): 15 g of Na_2_EDTA, 2.9 g of CaCl_2_, 9.2 g of ZnSO_4_·7H_2_O, 0.5 g of CuSO_4_, 0.43 g of MnSO_4_·H2O, 0.47 g of CoCl_2_, 0.48 g of Na_2_MoO_4_, and 5.1 g of FeSO_4_·7H2O. A biomass conversion ratio of 0.59 g/L per OD_600_ unit was determined by drying and weighing cells in predried Falcon tubes in a 100 °C oven overnight in triplicate.

### High-Pressure Liquid Chromatography Analysis by Ultraviolet Absorbance (HPLC-UV) and Mass Spectrometry (HPLC-MS)

Dopamine, hydroxytyrosol, tyrosol, 4-HPAC, 3,4-dHPAC, and (*S*)-reticuline were quantified by HPLC-UV using an Agilent 1200 HPLC system. Samples in 96-well plate format were diluted 1:2 with 100% acetonitrile (AcN) containing 0.1% trifluoroacetic acid (TFA), vortexed briefly, centrifuged for 5 min at 21,000 *g*, and then supernatant was analyzed. Supernatants from bioreactors were further diluted with 50% AcN/0.1% TFA as appropriate to stay within the range of the standard curves. Five μL of analyte was applied to an Eclipse XDB-C18 column (150 × 4.6 mm, 5 μm, Agilent Technologies) and separated using the following gradient at 1 mL/min: 0–10 min, 5–20% B; 10–15 min, 20–50% B; 15–15.1 min, 50–95% B; 15.1–25 min, 95% B; 25–28 min, 5% B where A was 0.1% TFA in water and B was 0.1% TFA in methanol. All compounds were detected at 280 nm.

Relative peak areas of BIAs and BIA-like scaffolds were assessed using an Agilent 6545 qTOF-MS. All samples were diluted 1:5 with 100% AcN containing 0.1% formic acid (FA) and water containing 0.1% FA was added to bring the final AcN concentration to 15%. Samples were centrifuged for 10 min at 4,000 *g*, and then supernatant was analyzed. Supernatants were diluted as necessary to avoid the saturation of the detector. Five μL of analyte was applied to a Zorbax Eclipse Plus C18 column (50 × 2.1 mm, 1.8 μm, Agilent Technologies) and separated using the following gradient at 0.3 mL/min flow rate: 0–4 min, 2–10% B; 4–6 min, 10–85% B; 6–7 min, 85% B, 7–7.1 min, 85–2% B where A was 0.1% FA in water and B was 0.1% FA in AcN. The column was reequilibrated for 2 min in 2% B at 0.45 mL/min. Settings: column compartment, 30 °C; sheath gas flow rate, 10 L/min; sheath gas temperature, 350 °C; drying gas flow rate, 12 L/min; drying gas temperature, 325 °C; nebulizing gas, 55 psig.

Dihydrosanguinarine pathway intermediates were assessed by HPLC-FT-MS using an Agilent 1290 Infinity II HPLC instrument (Agilent Technologies) and a 7T-LTQ-FT-ICR instrument (Thermo Fisher Scientific). Samples in 96-well plate format were extracted as per HPLC-qTOF-MS analysis. For determination of dihydrosanguinarine and sanguinarine concentration in bioreactors, fermentation broth was diluted 1:5 with 100% MeOH containing 0.1% HCl, and then further diluted to 1:100 with 100% MeOH/0.1% FA prior to centrifugation for 5 min at 4,000 *g*. Samples were diluted as necessary in 100% MeOH/0.1% FA to stay within the linear range of the MS. Five μL was applied to a Zorbax Eclipse Plus C18 column (50 × 2.1 mm, 1.8 μm, Agilent Technologies) and separated using the following gradient: 0.3 mL/min flow rate: 0–4 min, 2–10% B; 4–6 min, 10–85% B; 6–9 min, 85% B, 9–9.1 min, 85–2% B where A was 0.1% FA in water and B was 0.1% FA in AcN. The column was reequilibrated for 5 min in 2% B at 0.3 mL/min. Settings: scanning range, 100–400 *m*/*z*, resolution, 25,000; capillary voltage, 5 kV; source temperature, 350 °C.

Sources of HPLC-UV/MS reagents: water and acetonitrile, Fisher Scientific; methanol, Sigma-Aldrich; formic acid, Fluka; and trifluoroacetic acid, Sigma-Aldrich. Sources for authentic BIAs were: (*S*)-norcoclaurine, TRC Inc. (North York, Ontario, Canada); (*S*)-reticuline, gift from Dr. Peter Facchini; (*S*)-scoulerine, ChromaDex (Irvine, Ca, USA); (*S*)-stylopine, ChromaDex (Irvine, Ca, USA); protopine, TRC Inc.; sanguinarine, Sigma. Dihydrosanguinarine was derived from sanguinarine through sodium borohydride reduction.^55^

## Supplementary Material



## Data Availability

The data that support the findings of this study are available from the authors upon reasonable request.

## Notes

Supplemental Figure 1. Visualization of MAO localization by fluorescence microscopy. Supplemental Figure 2. Metabolite profile of strains expressing *MAO.* Supplemental Figure 3. Assessment of MAO peroxisomal compartmentalization. Supplemental Figure 4. Fed-batch fermentation of strain LP524. Supporting Information & Methods Supplementary Table 1. Strain lists Supplementary Table 2. Plasmid list Supplementary Table 3. Primer list Supplementary Table 4. Synthetic gene list (PDF)

## ⊥

Amyris Inc., Emeryville, California, USA 94608

## #

Department of Biology, Western University, London, Ontario, Canada N6A 3K7.

## ∇

Department of Chemical and Biological Engineering, Princeton University, Princeton, New Jersey, USA 08540.
