# Supplementary material for: Benzylisoquinoline Alkaloid Production in Yeast via Norlaudanosoline Improves Titer, Selectivity, and Yield
Source: ACS Synth Biol. 2026 Mar 4;15(3):1187–99. doi: 10.1021/acssynbio.5c00897 (PMC13010366; doi:10.1021/acssynbio.5c00897)
Supplement: Supplementary file 1 [file sb5c00897_si_001.pdf]

Supplemental Material

Benzylisoquinoline alkaloid production in yeast via norlaudanosoline improves titer, selectivity, and yield

Lauren Narcross [a,b,1], Michael E. Pyne [a,b,2], Kaspar Kevvai [a,b], Ka-Hei Siu [c,3], John E. Dueber [c,d] and Vincent J. J. Martin [a,b *]

[a] Department of Biology, Concordia University, Montréal, Québec, Canada H4B 1R6

[b] Centre for Applied Synthetic Biology, Concordia University, Montréal, Québec, Canada H4B 1R6

[c] Department of Bioengineering, University of California, Berkeley, Berkeley, California, USA 94720

[d] Biological Systems & Engineering Division, Lawrence Berkeley National Laboratory, Berkeley, California, USA 94720

[1] Present address: Amyris Inc., Emeryville, California, USA 94608

[2] Present address: Department of Biology, Western University, London, Ontario, Canada N6A 3K7

[3] Present address: Department of Chemical and Biological Engineering, Princeton University, Princeton, New Jersey, USA 08540

* Corresponding author: vincent.martin@concordia.ca

Supplemental Figures

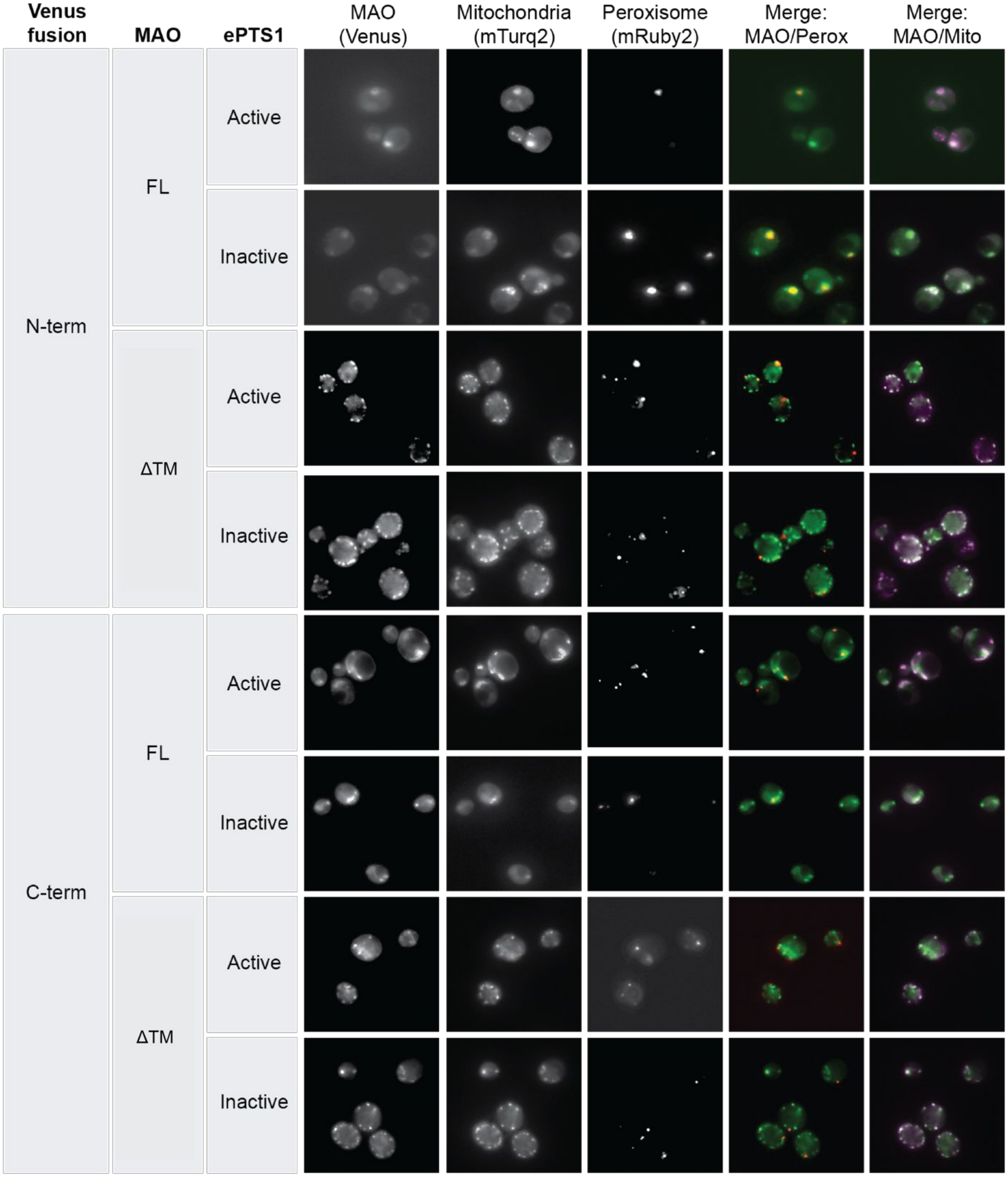

Supplemental Figure 1. Visualization of MAO localization by fluorescence microscopy. The fluorescent protein Venus was fused to the N-terminus (N-term) or C-terminus (C-term) of full-length MAO (FL) or MAOΔTM (ΔTM). At the extreme C-terminus of each fusion protein was an active ePTS1, targeting the cargo to the peroxisome, or an inactive ePTS1, expected to maintain cytosolic localization. Each fusion protein was expressed in strain yKSS001, which harbors *Su9ss-mTurq2* to visualize mitochondria and *mRuby-ePTS1* to visualize peroxisomes. Although mitochondrial morphology differs between strains expressing *MAO* and *MAOΔTM,* mitochondrial co-localization is observed throughout. Further, no differences are observed between MAO variants with active or inactive ePTS1.

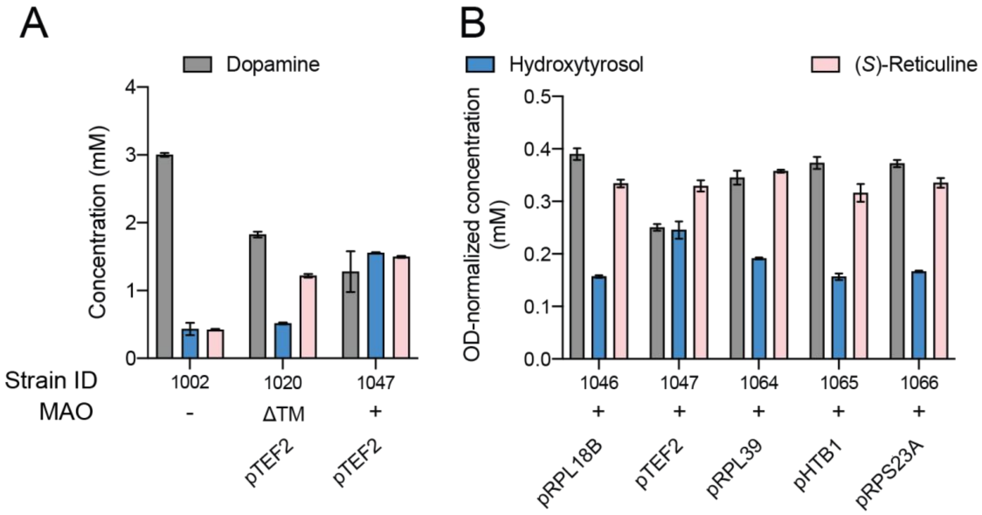

Supplemental Figure 2. Metabolite profile of strains expressing *MAO*. *MAO*, with (+) or without its C-terminal transmembrane helix (ΔTM), was integrated into strain LN1002 (*aro10*Δ) under a variety of promoter strengths. Strains were grown in synthetic complete media in deep well plates and then analyzed for dopamine, reticuline, and hydroxytyrosol content by LC-UV. Error bars refer to the mean and standard deviation of n = 2 biological replicates. (**A**) Strain LN1020, harboring MAOΔTM, produces more dopamine, less hydroxytyrosol, and less reticuline than strain LN1047, harboring full-length MAO. (**B**) Strains LN1064, LN1065, and LN1066, expressing *MAO* from the promoters of *RPL39*, *HTB1*, and *RPS23A*, respectively, have a metabolite profile that resembles strains LN1046 and LN1047. Metabolite content is represented normalized to final OD_{600}.

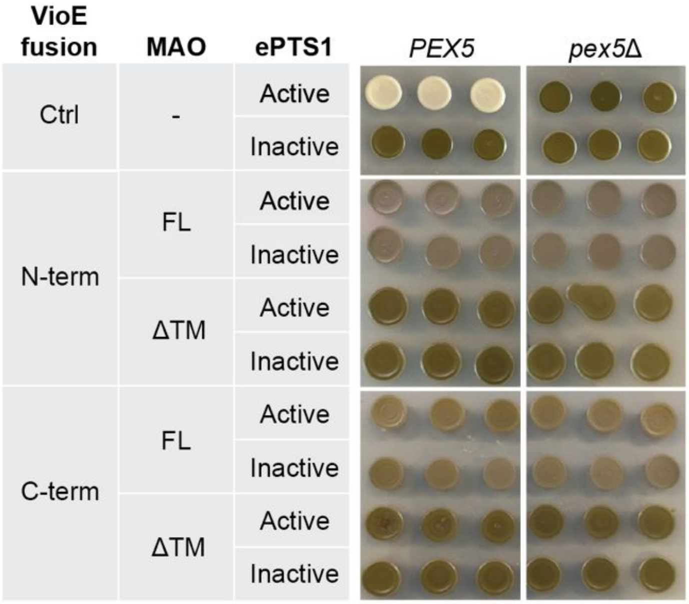

Supplemental Figure 1. Assessment of MAO peroxisomal compartmentalization. Agar spot assay to visualize efficiency of MAO import to the peroxisome. Strains yPSG335 (*PEX5*) and yPSG340 (*pex5*Δ) express the violacein pathway proteins VioA and VioB in the cytosol, which convert tryptophan to IPA imine dimer (colorless). VioE, fused to MAO or MAOΔTM at the N- or C-terminus, is required to convert IPA imine dimer to prodeoxyviolacein (green). Sequestration of VioE to the peroxisome, *via* active ePTS1, prevents the formation of colour (Ctrl, active ePTS1, *PEX5*). Inactive ePTS1 reduces peroxisomal import dramatically[1]; such strains should be a deep green (Ctrl, inactive ePTS1, *PEX5*). *pex5*Δ strains, defective in peroxisomal import, are also deep green. A lack of difference in colour between active and inactive ePTS1 fusion proteins indicates that MAO peroxisomal import is not effective. Expression of full-length MAO, especially as a VioE N-terminal fusion, results in a grey colour. Abbreviations: ePTS1, enhanced peroxisomal targeting sequence 1; Ctrl, control; FL, full-length; ΔTM, MAOΔTM; N-term, N-terminal enzyme fusion; C-term, C-terminal enzyme fusion.

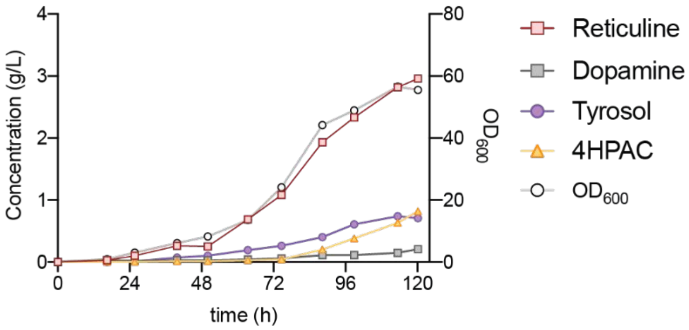

Supplemental Figure 2. Fed-batch fermentation of strain LP524. Strain LP524 was grown in a bioreactor using an exponential, sugar-limited feeding profile. Samples were regularly taken and assessed for biomass and for metabolite profile by LC-UV.

Supplemental Materials & Methods

Strain construction and growth media for MAO localization experiments

The *S. cerevisiae* strains for MAO localization and visualization experiments were BY4741 (*MATa his3Δ1 leu2Δ0 met15Δ0 ura3Δ0*) and BY4741 *pex5Δ*, ordered from Open Biosystems (GE Dharmacon). Wild-type yeast cultures were grown in YPD medium (10 g/L Bacto yeast extract, 20 g/L Bacto peptone, 20 g/L glucose). Selection of auxotrophic markers (*URA3*, *LEU2*, *HIS3*) was performed in synthetic complete (SC) medium (6.7 g/L Difco Yeast Nitrogen Base without amino acids (Spectrum Chemical); 2 g/L Drop-out Mix Synthetic minus appropriate amino acids, without Yeast Nitrogen Base (US Biological); 20 g/L glucose). All strains used are listed in Table S1.

Golden Gate assembly reactions were transformed into chemically competent *E. coli* prepared from strain TG1 (Lucigen). Transformed cells were selected on LB containing the antibiotics chloramphenicol, ampicillin or kanamycin.

Yeast expression vectors were built using Golden Gate assembly as described in the Yeast Toolkit (YTK) system[2]. Integration into the yeast genome via homologous recombination at the *URA3* or *LEU2* locus was achieved by transformation of linearized plasmids (*Not*I digestion, NEB). All transformations were performed using a standard lithium acetate method, and cells were plated onto selective auxotrophic SC agar plates with 2% glucose. Individual colonies were picked as biological replicates directly from this transformation plate and grown independently for further analysis.

Chromosomal integrations of the fluorescent localization reporters mRuby2-ePTS1 (peroxisome) and Su9(1-69)-mTurqoise2[3] (mitochondria) were performed by co-transforming a CEN6/ARS4 CRISPR plasmid (containing Cas9, a guide RNA for targeting the YMR206W locus

and a *HIS3* auxotrophic marker) with linearized repair DNA designed for homologous recombination. Cells were plated on histidine-dropout medium, re-streaked on histidine-dropout medium and then grown in non-selective medium to remove the CRISPR plasmid. Chromosomal integration was confirmed by PCR, and removal of CRISPR plasmid was confirmed by replica plating from non-selective medium onto histidine-dropout medium (a colony will not grow on histidine-dropout medium if the CRISPR plasmid has been removed).

Fluorescence microscopy

For MAO localization experiments, strains were grown to saturation in SC medium with 2% glucose and auxotrophic selection, diluted 50-fold into fresh selective medium and grown for 6–8 hrs. Cultures were resuspended in 1x PBS before spotting onto plain glass slides for imaging on a Zeiss Axio Observer D1 microscope with an X-Cite Series 120 fluorescent lamp, a Hamamatsu Orca-Flash 4.0 digital camera and ZEN 2.6 (blue edition) software. Images were analyzed using ZEN 3.0 (blue edition) software. The fluorescent protein variants used were CFP mTurqoise2, YFP Venus and RFP mRuby2.

Spot assays for prodeoxyviolacein production and visualization

Agar plate spots for visualization of prodeoxyviolacein production were generated by plating 5 µl of saturated culture on SC agar plates with 2% glucose and appropriate auxotrophic selection. Plates were grown at 30 °C and imaged at 48 hrs. Three biological replicates of each strain were plated and imaged.

Supplemental tables

Table S1. Strain lists

**Base strain for BIA synthesis**			
**Name**	**Brief description**	**Genotype**	**Ref.**
LP507	Strain derived from BY4741 capable of making 4.6 g/L reticuline. See reference for full detail.	*BY4741*. Oxidoreductase knockouts: Δ*aad3*, Δ*ydr541c*, Δ*ypr1*, Δ*adh6*, Δ*ari1*, Δ*gre2*, Δ*hfd1*. Mitochondrial fixes: *MIP1*, *CAT5*, *SAL1*. Aromatic amino acid flux: $ARO4^{FBR}$, $ARO7^{FBR}$, *ARO2*, *TYR1*. 4-HPAA synthesis: *ARO10*. Dopamine synthesis: *DODC*, *CYP76AD5*. Norcoclaurine synthesis: *CjNCSΔ35* (2 copies). Reticuline pathway: *Ps6OMT*, *PsCNMT*, *Ps4′OMT* (2 copies), *AtATR2*.	4

**Yeast strains generated for BIA synthesis**			
**Name**	**Parent**	**Brief Description**	**Genotype**
LP524	LP507	Complete second copy of reticuline pathway	**USERXII-1**::P_{TDH3}-*Ps*CNMT-T_{TDH1}-P_{CCW12}-*Ec*NMCH-T_{PGI1}
LN1002	LP524	Knockouts of native and overexpressed Aro10	Δ*Aro10*, Δ**FgF18**::P_{TDH3}-Aro10-T_{TDH1}
LN1044	LN1002	RNR2-MAO	**USERX-1**::P_{RNR2}-*Hs*MAO-A-T_{TDH1}
LN1046	LN1002	RPL18B-MAO	**USERX-1**::P_{RPL18B}-*Hs*MAO-A-T_{TDH1}
LN1047	LN1002	TEF2-MAO	**USERX-1**::P_{TEF2}-*Hs*MAO-A-T_{TDH1}
LN1049	LN1002	TDH3-MAO	**USERX-1**::P_{TDH3}-*Hs*MAO-A-T_{TDH1}
LN1020	LN1002	TEF2-MAOΔTM	**USERX-1**::P_{TEF2}-*Hs*MAO-AΔTM-T_{TDH1}
LN1015	LN1002	TEF2-MAOΔTM-p	**USERX-1**::P_{TEF2}-*Hs*MAO-AΔTM-ePTS1-T_{TDH1}
LN1063	LN1046	NCS-p	**USERX-3**::P_{TDH3}-*Cj*NCSΔ35-ePTS1-T_{ENO2}
LN1064	LN1002	RPL39-MAO	**USERX-1**::P_{RPL39}-*Hs*MAO-A-T_{TDH1}
LN1065	LN1002	HTP1-MAO	**USERX-1**::P_{HTB1}-*Hs*MAO-A-T_{TDH1}
LN1066	LN1002	RPS23A-MAO	**USERX-1**::P_{RPS23A}-*Hs*MAO-A-T_{TDH1}
LN1067	LN1064	NCS-p	**USERXI-2**::P_{TDH3}-*Cj*NCSΔ35-ePTS1-T_{ENO2}
LN1068	LN1065	NCS-p	**USERXI-2**::P_{TDH3}-*Cj*NCSΔ35-ePTS1-T_{ENO2}
LN1069	LN1066	NCS-p	**USERXI-2**::P_{TDH3}-*Cj*NCSΔ35-ePTS1-T_{ENO2}
LN1075	LN1015	BBE	**USERX-3**::P_{TDH3}-*Ec*BBE-T_{TDH1}
LN1078	LN1075	CFS/SPS/TNMT	**USERXI-2**::P_{PGK1}-*Sdi*CFS-T_{ADH1}-P_{TEF1}-*Ndo*SPS-T_{PGK1}-P_{TEF2}-TNMT-T_{ADH1}
LN1079	LN1075	BBE (2 copies)	**USERXI-2**::P_{TDH3}-*Ec*BBE-T_{TDH1}
LN1080	LN1079	CFS/SPS/TNMT	**USERIV-1**:: P_{PGK1}-*Sdi*CFS-T_{ADH1}-P_{TEF1}-*Ndo*SPS-T_{PGK1}-P_{TEF2}-TNMT-T_{ADH1}
LN1083	LN1080	MSH/P6H	**USERIX-1**::P_{PDC1}-P6H-T_{CYC1}-**linker**-P_{TDH3}-MSH-T_{ADH1}
LN1084	LN1080	MSH/P6H/TNMT	**USERIX-1**::P_{PDC1}-P6H-T_{CYC1}-**linker**-P_{TDH3}-MSH-T_{ADH1}-**linker**-P_{FBA1}-TNMT-T_{PGI1}

Yeast strains generated for assessment of MAO localization			
**Name**	**Parent**	**Plasmid used**	**Description**
yWCD230	BY4741	pWCD1351	Wild-type yeast (*his3*Δ)
yWCD231	BY4741 pex5Δ::KanMX	pWCD1351	Defective peroxisomal import (*his3*Δ)
yKSS001	yWCD230	pKS0864	YMR206WΔ::pHHF1-Su9(1-69)-mTurquoise2-tENO2-pTEF2-mRuby2-GSLGRGRR-SKL!-tPGK1
yKSS002	yKSS001	pKS0902	Microscopy: N-terminal VioE+Venus MAO, ePTS1
yKSS003	yKSS001	pKS0903	Microscopy: N-terminal VioE+Venus MAO, inactive ePTS1
yKSS004	yKSS001	pKS0904	Microscopy: N-terminal VioE+Venus MAOΔTM, ePTS1
yKSS005	yKSS001	pKS0905	Microscopy: N-terminal VioE+Venus MAOΔTM, inactive ePTS1
yKSS006	yKSS001	pKS0906	Microscopy: C-terminal VioE+Venus MAO, ePTS1
yKSS007	yKSS001	pKS0907	Microscopy: C-terminal VioE+Venus MAO, inactive ePTS1
yKSS008	yKSS001	pKS0908	Microscopy: C-terminal VioE+Venus MAOΔTM, ePTS1
yKSS009	yKSS001	pKS0909	Microscopy: C-terminal VioE+Venus MAOΔTM, inactive ePTS1
yPSG335	yWCD230	pJAS1052	pTDH3-VioA-tENO1-pTEF1-VioB-tPGK1
yPSG340	yWCD231	pJAS1052	pTDH3-VioA-tENO1-pTEF1-VioB-tPGK1
yKSS010	yPSG335	pKS0902	Violacein: N-terminal VioE+Venus MAO, ePTS1
yKSS011	yPSG335	pKS0903	Violacein: N-terminal VioE+Venus MAO, inactive ePTS1
yKSS012	yPSG335	pKS0904	Violacein: N-terminal VioE+Venus MAOΔTM, ePTS1
yKSS013	yPSG335	pKS0905	N-terminal VioE+Venus MAOΔSP, inactive ePTS1
yKSS014	yPSG335	pKS0906	Violacein: C-terminal VioE+Venus MAO, ePTS1
yKSS015	yPSG335	pKS0907	Violacein: C-terminal VioE+Venus MAO, inactive ePTS1
yKSS016	yPSG335	pKS0908	Violacein: C-terminal VioE+Venus MAOΔTM, ePTS1
yKSS017	yPSG335	pKS0909	Violacein: C-terminal VioE+Venus MAOΔTM, inactive ePTS1
yKSS018	yPSG340	pKS0902	Violacein, import defects: N-terminal VioE+Venus MAO, ePTS1
yKSS019	yPSG340	pKS0903	Violacein, import defects: N-terminal VioE+Venus MAO, inactive ePTS1
yKSS020	yPSG340	pKS0904	Violacein, import defects: N-terminal VioE+Venus MAOΔTM, ePTS1
yKSS021	yPSG340	pKS0905	Violacein, import defects: N-terminal VioE+Venus MAOΔTM, inactive ePTS1
yKSS022	yPSG340	pKS0906	Violacein, import defects: C-terminal VioE+Venus MAO, ePTS1
yKSS023	yPSG340	pKS0907	Violacein, import defects: C-terminal VioE+Venus MAO, inactive ePTS1
yKSS024	yPSG340	pKS0908	Violacein, import defects: C-terminal VioE+Venus MAOΔTM, ePTS1
yKSS025	yPSG340	pKS0909	Violacein, import defects: C-terminal VioE+Venus MAOΔTM, inactive ePTS1
yKSS026	yPSG335	pKS1124	Violacein: VioE-Venus fusion, ePTS1
yKSS027	yPSG335	pKS1125	Violacein: VioE-Venus fusion, inactive ePTS1
yKSS028	yPSG340	pKS1124	Violacein, import defects: VioE-Venus fusion, active ePTS1
yKSS029	yPSG340	pKS1125	Violacein, import defects: VioE-Venus fusion, inactive ePTS1

Table S2. Plasmid list

Plasmids		
**Name**	**Brief Description**	**Ref.**
pCAS-G418	Yeast replicative plasmid (2μ) with Cas9 and $tRNA^{Tyr}$-driven gRNA. G418 resistance.	[5]
pCAS-Hyg	Yeast replicative plasmid (2μ) with Cas9 and $tRNA^{Tyr}$-driven gRNA. Hyg resistance.	[4]
pBSC011	P_{TDH3}-CjNCSΔ35-T_{ENO2}	[4]
pGC1899	Harbors CFS, SPS, TNMT for integration into genome	This work
pGC997	Harbors MSH, P6H, TNMT for integration into genome	[6]
pHUM	Harbors *HIS3*, *URA3*, *MET17* for prototrophy restoration of S288C-derived strains	[7]
pWCD1351	*HIS3*	This work
pKS0864	Integrative plasmid harbouring pHHF1-Su9ss-mTurquoise2-tENO2-pTEF2-mRuby2-GSLGRGRR-SKL!-tPGK1 and a *HIS3* marker	This work
pKS0902	pTEF2-VioE-Venus-Hs_MAO-A-ePTS1-tADH1	This work
pKS0903	pTEF2-VioE-Venus-Hs_MAO-A-dead_PTS1-tADH1	This work
pKS0904	pTEF2-VioE-Venus-Hs_MAO-A(1-497)-ePTS1-tADH1	This work
pKS0905	pTEF2-VioE-Venus-Hs_MAO-A(1-497)-dead_PTS1-tADH1	This work
pKS0906	pTEF2-Hs_MAO-A-VioE-Venus-ePTS1-tADH1	This work
pKS0907	pTEF2-Hs_MAO-A-VioE-Venus-dead_PTS1-tADH1	This work
pKS0908	pTEF2-Hs_MAO-A(1-497)-VioE-Venus-ePTS1-tADH1	This work
pKS0909	pTEF2-Hs_MAO-A(1-497)-VioE-Venus-dead_PTS1-tADH1	This work
pKS1124	pTEF2-VioE-Venus-ePTS1-tADH1	This work
pKS1125	pTEF2-VioE-Venus-dead_PTS1-tADH1	This work

Table S3. Primer list

Yeast strains			
**Number**	**Name**	**Sequence (5′->3′)**	**Description**
LB33	HDVgRNA_F	CACCTATATCTGCGTGTTGC	When paired: amplify empty guide RNA transcription cassette from pCAS-G418/Hyg. Use with two internal primers providing guide RNA target sequence.
LB34	gRNA_scaffold_R	GTCAAGACTGTCAAGGAGG	
LB1968	X-1_UP_F	cgctcactagtagacaacacacg	Amplify X-1 upstream homology region with homology to gene cassettes
LB1969	(LV3)_X-1_UP_R	gcatttttattatataagttgttttattcagagtattcc tggaagcatacagaatattcactaac	
LB1970	(LV5)_X-1_DN_F	Cctctttatattacatcaaaataagaaaataattat aacaccacgattgagtgtctgcactcttattc	Amplify X-1 downstream homology region with homology to gene cassettes
LB1971	X-1_DN_R	cctttttccaattcttaggctatttgg	
LB1964	X-1_guide_F	gtagctacaagaacatatggGTTTTAGA GCTAGAAATAGCAAGT	Use with LB33/LB34 to introduce X-1 guide sequence to CRISPR plasmid
LB1965	X-1_guide_R	ccatatgttcttgtagctacAAAGTCCCA TTCGCCACCCGAA	
LB1987	X-3_UP_F	ggctactgattttgttaagcaactc	Amplify X-3 upstream homology region with homology to gene cassettes
LB1979	(LV3)_X-3_UP_R	gcatttttattatataagttgttttattcagagtattcc tgagaagaaattttgggggtaatatg	
LB1980	(LV5)_X-3_DN_F	Cctctttatattacatcaaaataagaaaataattat aacagggaaataaggtttaaaggcactg	Amplify X-3 downstream homology region with homology to gene cassettes
LB1981	X-3_DN_R	ggtatctcaatgaacgagctcg	

LB1974	X-3_guide_F	gatcgccgaatggcacgcgaGTTTTAGAGCTAGAAATAGCAAGT	Use with LB33/LB34 to introduce X-3 guide sequence to CRISPR plasmid
LB1975	X-3_guide_R	tcgcgtgccattcggcgatcAAAGTCCCATTCGCCACCCGAA	
LB1988	XI-2_UP_F	ggtttctgaaaaaagaagtagtcg	Amplify XI-2 upstream homology region with homology to gene cassettes
LB1989	(LV3)_XI-2_UP_R	gcatttttattatataagttgttttattcagagtattcctctgaaagcgctagtcgtgtgtacc	
LB1990	(LV5)_XI-2_DN_F	Cctctttatattacatcaaaataagaaaataattataacagctttgcagttttcgtggctag	Amplify XI-2 downstream homology region with homology to gene cassettes
LB1991	XI-2_DN_R	ctataacatggtttacaaacccgagg	
LB1984	XI-2_guide_F	actttgtcgtttcttactttGTTTTAGAGCTAGAAATAGCAAGT	Use with LB33/LB34 to introduce XI-2 guide sequence to CRISPR plasmid
LB1985	XI-2_guide_R	aaagtaagaaacgacaaagtAAAGTCCCATTCGCCACCCGAA	
LN1100	IV-1_UP_F	CGTGCGCTTGAGATTCAGT	Amplify IV-1 upstream homology region with homology to gene cassettes
LN1101	(LV3)_IV-1_UP_R	gcatttttattatataagttgttttattcagagtattcctAGAGTTCCCGTCGGAAT	
LN1102	(LV5)_IV-1_DN_F	cctctttatattacatcaaaataagaaaataattataacaCGTTACTAGCGTTGCAAGTGG	Amplify IV-1 downstream homology region with homology to gene cassettes
LN1103	IV-1_DN_R	GGATTTGGTTTAGCAGCAGTC	
LN1106	IV-1_guide_F	CGCCGGCTGGGCAACACCTTCGGGTGGCGAATGGGACTTTCTGCAAGGAAGTTTAAGCGT	Introduce IV-1 guide RNA sequence to CRISPR plasmid using overlap extension
LN1107	IV-1_guide_R	GACTAGCCTTATTTTAACTTGCTATTTCTAGCTCTAAAACACGCTTAAACTTCCTTGCAGA	
LN1092	II-1_UP_F	GCGTTCACAGTTACTCTTTTAGAAC	Amplify II-1 upstream homology region with homology to gene cassettes
LN1093	(LV3)_II-1_UP_R	gcatttttattatataagttgttttattcagagtattcctAAAATAACATGTTGCGTGCAC	
LN1094	(LV5)_II-1_DN_F	cctctttatattacatcaaaataagaaaataattataacaGACAAACTTTACAAAGAAGACACCC	Amplify II-1 downstream homology region with homology to gene cassettes
LN1095	II-1_DN_R	GTATGCCGTGATATGAACAAAC	
LN1098	II-1_guide_F	CGCCGGCTGGGCAACACCTTCGGGTGGCGAATGGGACTTTAACTGCTCAGGGCGGATAAC	Introduce II-1 guide RNA sequence to CRISPR plasmid using overlap extension
LN1099	II-1_guide_R	GACTAGCCTTATTTTAACTTGCTATTTCTAGCTCTAAAACGTTATCCGCCCTGAGCAGTT	
LN1108	IX-1_UP_F	CAACTGCTAAGAACTCTGTGATCTTC	Amplify IX-1 upstream homology region with homology to gene cassettes
LN1109	(LV3)_IX-1_UP_R	gcatttttattatataagttgttttattcagagtattcctTCGCGAGATAGAACGACATC	
LN1110	(LV5)_IX-1_DN_F	cctctttatattacatcaaaataagaaaataattataacaTTGATGACACTAGCGGACTTG	Amplify IX-1 downstream homology region with homology to gene cassettes
LN1111	IX-1_DN_R	GGCAGAAAACTACCCGTAGAATAC	
LN1114	IX-1_guide_F	CGCCGGCTGGGCAACACCTTCGGGTGGCGAATGGGACTTTAT	

		CTTAAATGAAAGACAGAGGTTTT	
LN1115	IX-1_guide_R	GACTAGCCTTATTTTAACTTGCTATTTCTAGCTCTAAAACCTCTGTCTTTCATTTAAGATAAAG	Introduce IX-1 guide RNA sequence to CRISPR plasmid using overlap extension
LN14	(LV3)_pRNR2_F	aggaatactctgaataaaacaacttatataataaaaatgcAGTCGAACAAGAAGCAGG	Amplify pRNR2 with homology to gene cassettes
LN456	pRNR2_R	GGTAATTGGACAAATAAATACGTGTATTAAG	
LN18	(LV3)_pRPL18B_F	aggaatactctgaataaaacaacttatataataaaaatgcaagaggatgtccaatatttt	Amplify pRPL18B with homology to gene cassettes
LN455	pRPL18B_R	tttgtttttgttttcttctaattgatt	
LN1080	(LV3)_pRPL39_F	aggaatactctgaataaaacaacttatataataaaaatgcCTTGGATATGTATGTTGGTCTTGTT	Amplify pRPL39A with homology to gene cassettes
LN1081	pRPL39_R	GTTGATCTATCTGTGTTTATTTGCTTG	
LN1083	(LV3)_pHTB1_F	aggaatactctgaataaaacaacttatataataaaaatgcATGATGGTTCAACAAGACCAGA	Amplify pHTB1 with homology to gene cassettes
LN1084	pHTB1_R	TGTATGTGTGTATGGTTTATTTGTGG	
LN1086	(LV3)_pRPS23A_F	aggaatactctgaataaaacaacttatataataaaaatgcGTCGGTCGCACTAGACTTTTC	Amplify pRPS23A with homology to gene cassettes
LN1087	pRPS23A_R	CTTTGTTTATTTCCTGTTGTCTTAGG	
LN27	(LV3)_pTEF2_F	aggaatactctgaataaaacaacttatataataaaaatgcttgataggtcaagatcaatgtaaac	Amplify pTEF2 with homology to gene cassettes
LN454	pTEF2_R	gtttagttaattatagttcgttgaccg	
PP120	(LV3)_pTDH3_F	AGGAATACTCTGAATAAAACAACTTATATAATAAAAATGCtcgagtttatcattatcaatact	Amplify pTDH3 with homology to gene cassettes
LN398	pTDH3_R	TTTGTTTGTTTATGTGTGTTTATTcg	
LB1130	tTDH1_F	ATAAAGCAATCTTGATGAGGATAATG	Amplify tTDH1 with homology to gene cassettes
LB1131	(LV5)_tTDH1_R	tgttataattattttcttattttgatgtaatataaagagGCCATCCTAGAACTTCAATTCACCAC	
LN247	tPGI1_F	aacaaatcgctcttaaatatatacc	Amplify tPGI1 with homology to gene cassettes
PP127	(LV5)_tPGI1_R	tgttataattattttcttattttgatgtaatataaagaggggtatactggaggcttcat	
LN1018	Aro10g1_OE_F	CGCCGGCTGGGCAACACCTTCGGGTGGCGAATGGGACTTTATATTCGCCTTGTGGACACT	Introduce guide RNA targeting Aro10 to CRISPR plasmid using overlap extension
LN1019	Aro10g1_OE_R	GACTAGCCTTATTTTAACTTGCTATTTCTAGCTCTAAAACAGTGTCCACAAGGCGAATAT	
LN1020	Aro10_repair_OE_F	ATTGCCGAGGTCATGCTGAGCATTTGTCGTACTTTTGTGCCGTATATTAAAG	Generate repair template for native Aro10 knockout using overlap extension

LN1021	Aro10_repair_OE_R	AAAGAACTCTGTGGTAGTGGTAAAATAGCTTTAATATACGGCACAAAAGTACGA	
LN1022	FgF18_repair_OE_F	tttcagtagatttggtaactgtgcaaccataactcatgccaatcgtc	Generate repair template for heterologous Aro10 knockout using overlap extension
LN1023	FgF18_repair_OE_R	tgtgatgaattttgagagcccacttttgttggggacgattggcatgagttatg	
LN459	(pRNR2)_HsMAO-A_F	GAATCCAAACTTAATACACGTATTTATTTGTCCAATTACCATGGAAAATCAAGAAAAGGCATC	Amplify *Hs*MAO-A with homology to pRNR2
LN458	(pRPL18B)_HsMAO-A_F	atagaaagaaaaaatcaattagaagaaaacaaaaaacaaaATGGAAAATCAAGAAAAGGCATC	Amplify *Hs*MAO-A with homology to pRPL18B
LN1089	(pRPL39)_HsMAO-A-F	AATTCGAAAAAGACAAGCAAATAAACACAGATAGATCAACATGGAAAATCAAGAAAAGGCATC	Amplify *Hs*MAO-A with homology to pRPL39
LN1090	(pHTB1)_HsMAO-A_F	ATAGACAAGTCAAACCACAAATAAACCATACACACATACAATGGAAAATCAAGAAAAGGCATC	Amplify *Hs*MAO-A with homology to pHTB1
LN1091	(pRPS23A)_HsMAO-A_F	AAATTTTACAAAAACCTAAGACAACAGGAAATAAACAAAGATGGAAAATCAAGAAAAGGCATC	Amplify *Hs*MAO-A with homology to pRPS23A
LN457	(pTEF2)_HsMAO-A_F	tttttagaatatacggtcaacgaactataattaactaaacATGGAAAATCAAGAAAAGGCATC	Amplify *Hs*MAO-A with homology to pTEF2
LN396	(pTDH3)_HsMAO-A_F	caagaacttagtttcgaATAAACACACATAAACAAACAAAATGGAAAATCAAGAAAAGGCATC	Amplify *Hs*MAO-A with homology to pTDH3
LN397	(tTDH1)_HsMAO-A_FL_R	attcaaaaaaaaatcattatcctcatcaagattgctttatTTATGATCTTGGCAACAATTTGTAC	Amplify full-length *Hs*MAO-A with homology to tTDH1
LN978	(ePTS1)_HsMAO-A_dSP_R	CAACTTAGAACGACGACCACGACCTAATGATGGCAAGTTTCTTTCCCAAAAaGTATGAGTGATTTCAAC	Amplify signal peptide-less *Hs*MAO-A with C-terminal ePTS1 tag
LN979	(ePTS1)_TDH1t_F	TTAGGTCGTGGTCGTCGTTCTAAGTTGTAAataaagcaatcttgatgaggataatg	Amplify tTDH1 with homology to C-terminal ePTS1 tag
LN1071	(pTEF1)_CjNCS_F	cttcttgctcattagaaagaaagcatagcaatctaatctaagttttaatAAACAAtggaagaaactgtaatgttatatc	Amplify *Cj*NCS with homology to pTEF1
LN1072	(ePTS1)_CjNCS_R	ttaCAACTTAGAACGACGACCACGACCTAActcagaagatttgtgcttattttc	Amplify *Cj*NCS with C-terminal ePTS1 tag
LN1070	(ePTS1)_tPGI1_F	TTAGGTCGTGGTCGTCGTTCTAAGTTGtaaaacaaatcgctcttaaatatatacctaaag	Amplify tPGI1 with N-terminal ePTS1 tag
LN564	(pTDH3)_EcBBE_F	caagaacttagtttcgaATAAACACACATAAACAAACAAAATGGAAAATAAGACACCGATTTTC	Amplify BBE with homology to pTDH3/tPGI1

LN565	(tPGI1)_EcBBE_R	ctttaatgttctttaggtatatatttaagagcgatttgttTTATATAACGACTTCTCCCCCG	
PP118	(LV3)_pPDC1_F	AGGAATACTCTGAATAAAACAACTTATATAATAAAAATGCacatgcgactgggt	Amplify genes from pGC997 with homology to integration sites
PP124	(LV5)_tADH1_R	TGTTATAATTATTTTCTTATTTTGATGTAATATAAAGAGGgcatgccggtagag	Amplify MSH, P6H from pGC997 with homology to integration sites
PP127	(LV5)_tPGI1_R	TGTTATAATTATTTTCTTATTTTGATGTAATATAAAGAGGggtatactggaggcttcat	Amplify MSH, P6H, and TNMT from pGC997 with homology to integration sites
LN1124	SPS-FP	gcatcgtctcatcggtctcatatggaagaatctttctggattgtctcc	Amplify stylopine synthase for Golden Gate cloning
LN1125	SPS-RP	atgccgtctcaggtctcaggatctaataaccgttaatagatggagaacct	
LN1126	CFS-FP	gcatcgtctcatcggtctcatatggaagaatctttctggttggttacc	Amplify cheilanthifoline synthase for Golden Gate cloning
LN1127	CFS-RP	atgccgtctcaggtctcaggatctaatgagttcttggggtgatcttag	
LN1128	TNMT-FP	gcatcgtctcatcggtctcatatgggttcaatagatgaggtcaagaag	Amplify TNMT for Golden Gate cloning
LN1129	TNMT-RP	atgccgtctcaggtctcaggatctacttcttcttgaaaagcagctgc	

Table S4. Synthetic gene list

HsMAO-A
atggaaaatcaagaaaaggcatctattgcaggtcacatgtttgatgttgttgttataggtggaggaatttcaggtttgtcagctgctaaattgttaa
ctgagtacggtgtttctgttttagtattagaggctagagacagagttggtggtagaacctatacaatcagaaacgaacatgtagactacgttga
cgttggtggagcttatgttggtcctactcagaataggattttaaggttgtctaaggaattaggtattgaaacttataaagttaatgtttcagaaaga
ttagtacagtatgttaaaggtaagacatatccatttagaggtgcctttccaccagtttggaatcctatcgcttacttggattacaataatttgtggag
aaccattgataatatgggtaaggagatcccaactgacgcaccttgggaggcacagcatgcagataagtgggacaagatgaccatgaagga
attaatcgataaaatatgttggactaaaaccgcaaggagattcgcctatttatttgtaaatattaacgtaacttctgaacctcatgaagtttcagca
ttgtggttcttatggtatgtcaagcagtgtggtggtacaacaagaatattttcagttacaaacggtggtcaagaaagaaaattcgtcggtggttct
ggacaggtatctgaaagaattatggatttattaggtgatcaagtcaaattgaatcatccagttacccatgtagaccaatcttctgataacatcatc
atagaaactttaaatcatgaacattatgagtgtaaatatgtcataaacgcaattcctccaaccttgactgccaaaatccattttagacctgaattac
ctgctgaaagaaaccaattaatccaaagattgccaatgggtgctgtaatcaaatgtatgatgtattacaaagaagctttctggaagaagaaag
attactgtggttgcatgattatagaggatgaagatgctccaatttctatcaccttagacgatactaaacctgatggatctttacctgccataatgg
gattcatattggccagaaaggccgatagattggcaaagttacataaagaaattagaaagaagaaaatctgtgagttatatgctaaagtcttagg
ttctcaagaagccttgcatcctgtccattatgaagaaaagaactggtgtgaggaacaatattctggaggatgttacactgcttatttcccacctg
gaattatgacacaatacggaagagttatcaggcagccagtcggtagaattttctttgcaggaactgaaaccgctacaaaatggtctggttatat
ggaaggtgcagtagaggctggtgaaagggctgctagagaagttttaaatggattaggaaaagtcactgaaaaggatatttgggtacaggag
cctgagtctaaagatgttcctgctgttgaaatcactcataccttttgggaaagaaacttgccatcagtctcaggtttgttgaaaatcattggtttctc
tacctcagtcaccgccttgggattcgttttgtataagtacaaattgttgccaagatca

*EcBBE (*from *Eschscholzia californica*)
atggaaaataagacaccgattttcttttcattatcaatcttcctatcattgctaaattgcgcgcttggcgggaatgatctattgtcatgcctaacgtt
caatggtgtacgtaatcatactgtgtttagcgccgacagtgacagtgacttcaataggtttcttcaccttagtatacaaaaccctttgttccaaaa
ctcactaatatccaagcctagtgctattatccttccggggagcaaagaggagttatccaacactataagatgtataagaaaaggttcctggacc
ataaggctgaggtcaggaggtcattcctacgaaggtctgtcatacaccagtgacacacccttcattcttatagacttaatgaatctaaatagggt
aagtattgatttagaatcagaaacagcgtgggttgagagcggatcaactctaggagaattatattacgcgataacggaaagttcttcaaagctt
ggatttaccgcagggtggtgccctaccgtgggtacggggggtcacatctccgggggggctttggcatgatgagtagaaagtatgggcta
gctgctgacaatgtagtagatgcgatattaatcgacgcgaatggggctatcttagacaggcaagctatgggagaggacgtgttttgggccatt
agaggcggtggcggtggggtatggggagccatatacgcctggaaaataaagcttctacctgtccccgaaaaagtgaccgtattccgtgtaa
ctaaaaacgtggcgatagacgaggcgacatcactgctacacaagtggcagttcgtcgctgaagaattggaagaggactttactctgtccgtt
ctgggcggggcagatgagaagcaggtatggttgacgatgttaggctttcactttggactgaaaacagtagcaaagtcaacctttgatttacttt
tccctgaactaggacttgtagaggaggattaccttgaaatgtcctgggggaatccttcgcgtatctagctggattggaaaccgtgagccaac
ttaataatagattcctgaagtttgatgaaagggcattcaaaaccaaagttgatttaacgaaggagccgctgcctagcaaagcgttttatggtctt
ctggagagattatctaaagagccaaatggtttcatagctttgaacgggttcgggggacaaatgtcaaagatctcctcagacttcaccccgttcc
ctcatcgttccggtacgaggttgatggtagaatacatcgtggcatggaatcaatcagaacaaaagaagaaaaccgagtttcttgactggttgg
aaaaagtttacgaatttatgaaaccgtttgtatctaagaatccccgtttgggatacgtcaaccatatagatttagatcttgggggaatagactgg
gggaataagacagtagttaacaatgcgatagagatcagtcgttcatggggtgagtcatatttttttgtccaattacgagagattaatccgtgctaa
aaccctaatagatcccaataatgtgtttaatcacccgcagtccatccccccgatggccaattttgattaccttgagaagacacttgggtccgac
gggggagaagtcgttatataa

References

(1) DeLoache, W. C.; Russ, Z. N.; Dueber, J. E. Towards repurposing the yeast peroxisome for compartmentalizing heterologous metabolic pathways. *Nature communications* **2016**, *7* (1), 11152.

(2) Lee, M. E.; DeLoache, W. C.; Cervantes, B.; Dueber, J. E. A highly characterized yeast toolkit for modular, multipart assembly. *ACS Synthetic Biology* **2015**, *4* (9), 975-986.

(3) Westermann, B.; Neupert, W. Mitochondria-targeted green fluorescent proteins: convenient tools for the study of organelle biogenesis in Saccharomyces cerevisiae. *Yeast* **2000**, *16* (15), 1421-1427.

(4) Pyne, M. E.; Kevvai, K.; Grewal, P. S.; Narcross, L.; Choi, B.; Bourgeois, L.; Dueber, J. E.; Martin, V. J. A yeast platform for high-level synthesis of tetrahydroisoquinoline alkaloids. *Nature Communications* **2020**, *11* (1), 1-10.

(5) Ryan, O. W.; Skerker, J. M.; Maurer, M. J.; Li, X.; Tsai, J. C.; Poddar, S.; Lee, M. E.; DeLoache, W.; Dueber, J. E.; Arkin, A. P. Selection of chromosomal DNA libraries using a multiplex CRISPR system. *Elife* **2014**, *3*, e03703.

(6) Fossati, E.; Ekins, A.; Narcross, L.; Zhu, Y.; Falgueyret, J.-P.; Beaudoin, G. A. W.; Facchini, P. J.; Martin, V. J. J. Reconstitution of a 10-gene pathway for synthesis of the plant alkaloid dihydrosanguinarine in *Saccharomyces cerevisiae*. *Nature Communications* **2014**, *5*, 3283.

(7) Mülleder, M.; Campbell, K.; Matsarskaia, O.; Eckerstorfer, F.; Ralser, M. *Saccharomyces cerevisiae* single-copy plasmids for auxotrophy compensation, multiple marker selection, and for designing metabolically cooperating communities. *F1000Research* **2016**, *5*.
